# Host–Guest Interactions of Ruthenium(II) Arene Complexes with Cucurbit[7/8]uril

**DOI:** 10.1021/acs.inorgchem.4c01755

**Published:** 2024-07-17

**Authors:** Elisa Roth, Risnita Vicky Listyarini, Thomas S. Hofer, Monika Cziferszky

**Affiliations:** † Institute for Pharmacy, Pharmaceutical Chemistry, Department of Chemistry and Pharmacy, University of Innsbruck, Innrain 80/82, A-6020 Innsbruck, Austria; ‡ Institute of General, Inorganic and Theoretical Chemistry, Center for Chemistry and Biomedicine, University of Innsbruck, Innrain 80-82, A-6020 Innsbruck, Austria; § Chemistry Education Study Program, Sanata Dharma University, Yogyakarta 55282, Indonesia

## Abstract

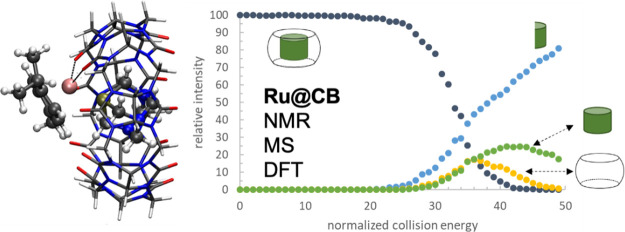

Cucurbit[*n*]urils (CB[*n*]s) have been recognized for their chemical and thermal stability, and their ability to bind many neutral and cationic guest molecules makes them excellent hosts in a range of supramolecular applications. In drug delivery, CB[*n*]s can enhance drug solubility, improve chemical and physical drug stability, and allow for triggered and controlled release. This study aimed to investigate the ability of CB[7] and CB[8] as molecular hosts to bind ruthenium(II) arene complexes that are current anticancer lead structures in the area of metallodrugs. Both, experimental and computational methods, led to insights into the binding preferences and geometries of [Ru^II^(cym)Cl_2_]_2_ (**1**; cym = η^6^-*p*-cymene), [Ru^II^(cym)(dmb)Cl_2_]) (**2**; cym = η^6^-*p*-cymene; dmb = 1,3-dimethylbenzimidazol-2-ylidene), and [Ru^II^(cym)(pta)Cl_2_] (**3**, RAPTA-C; cym = η^6^-*p*-cymene; pta = 1,3,5-triaza-7-phospha-adamantane) with CB[7] and CB[8]. Competition experiments by mass spectrometry revealed clear preferences of **2** for CB[8] and **3** for CB[7]. Based on a comparison of the associated interaction energies from quantum chemical calculations as well as experimental data, **3**@CB[7] clearly prefers a binding mode, where the pta ligand is located inside the cavity of the host, and the metal ion interacts with two of the carbonyl groups on the rim of CB[7]. In contrast, complex **2** binds in two different orientations with interaction energies similar to those of both CB[*n*]s, with the cym ligand being either inside or outside of the cavity. These findings suggest that ruthenium(II) arene complexes are able to form stable host–guest interactions with CB[*n*]s, which can be exploited as drug delivery vehicles in further metallodrug development to improve their chemical stability.

## Abstract 2

Two ruthenium(II) arene complexes with anticancer properties as well as their ruthenium(II) precursor were tested for their ability to form inclusion complexes with cucurbiturils CB[7] and CB[8]. Binding modes and preferences were analyzed by NMR, HR-ESI−MS, and theoretical calculations. Hydrolyzed and charged ruthenium complexes formed inclusion complexes with the ruthenium ion engaging in ion dipole interactions with the carbonyl-laced rim of CB[*n*] and one ligand immersed into the cavity.

## Introduction

Advances in cancer research and treatment have led to improved outcomes for many patients, however, malignant tumors remain a leading cause of death. For a large fraction of cancer patients, the three worldwide approved platinum(II)-based chemotherapeutic agents cisplatin, oxaliplatin, and carboplatin are still in use almost 50 years after cisplatin’s first approval. Often, these drugs are used in combination with other chemotherapeutics and treatment options. The severe and dose-limiting side effects of platinum-based drugs are well-known. As a consequence, ample funds and efforts were and are still directed toward the development of improved treatment options for cancer patients. Immunotherapy,^1^ photodynamic therapy,^2^ and targeted therapy using monoclonal antibodies^3^ have been successfully implemented in the clinic over the last two decades.

In the field of metal-based chemotherapeutic agents,^4^ the focus is on the development of more efficient and targeted compounds with better toxicity profiles as well as targeted delivery.^5^ However, only a few metal-based drug candidates have entered clinical trials since the approval of the above-mentioned platinum agents. For the ruthenium(III)-based compound BOLD-100, promising efficacy and safety data were published recently.^6^

Organometallic complexes of ruthenium(II) moved onstage for anticancer therapy in the 1990s.^7,8^ Sadler and Dyson independently started to investigate half-sandwich structures with π-bound ligands, the so-called “piano-stool” compounds. Sadler and co-workers synthesized chelating ethylenediamine-based ruthenium(II) arene complexes (READ),^9,10^ while Dyson and co-workers prepared monodentate triaza-phosphaadamantane ruthenium(II) arene complexes (RAPTA),^11,12^ which have been shown to exhibit antiproliferative, antiangiogenic, and antimetastatic properties. Both stimulated a lot of research in these structural scaffolds.^12,13^ Their modes-of-action differ from cisplatin’s and its analogues as DNA does not seem to be the main molecular target. Recent advancements in target identification strategies^14^ helped identify pleiotrophin, midkine, and histone proteins as molecular targets of RAPTA compounds^15^ as well as plectin for another ruthenium(II) arene complex called plecstatin.^16^ Also, NHC-ligands have been employed for the development of anticancer agents based on organoruthenium half-sandwich compounds.^17^

**Figure 1:**
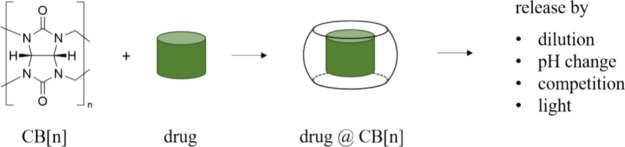
General concept of using CB[*n*] for drug delivery.

In contrast to organic small-molecule drugs, many metal-based drugs are capable of forming one or more dative covalent bonds with their target or, in fact, other suitable nucleophiles encountered en route to the target. This feature seems to be the underlying cause of many of the severe side effects of cisplatin and others.^18^ The design and synthesis of sophisticated delivery systems for reactive species such as cisplatin is a viable solution for the problem. A number of recent reviews^5,19−21^ showcase the multitude of ideas and options to deliver anticancer metallodrugs in a safe and targeted way. The use of macrocyclic hosts, in particular cucurbiturils (CB[*n*]s), holds promise as highly biocompatible receptors able of encapsulating therapeutic agents noncovalently and releasing them by an appropriate stimulus (see Figure 1).^22^ Their thermal and chemical stability, the availability of different sizes, their ability to bind a plethora of cationic and neutral compounds with high affinities (*K*_a_ > 10^4^ M^–1^),^23^ and their nontoxicity make them attractive macrocyclic hosts for applications in medicinal chemistry.^24,25^

The encapsulation of a platinum anticancer drug into a macrocyclic host was first reported by Kim and co-workers^26^ for oxaliplatin and CB[7], which formed a 1:1 inclusion complex, resulting in enhanced stability of the platinum drug. Later, Chen et al.^27^ showed increased antitumor activity of oxaliplatin@CB[7] in cancer cells that overexpressed spermine. On one hand, spermine led to competitive replacement of oxaliplatin and release of the drug. On the other side, CB[7] consumed some of the overexpressed spermine, which is essential for (tumor) cell growth and proliferation. In healthy cells, this supramolecular construct showed significantly reduced cytotoxicity compared with free oxaliplatin.

Cisplatin was shown to form a 1:1 inclusion complex with CB[7] with the platinum atom and both chlorido ligands located inside the cavity of the macrocycle, stabilized by hydrogen bonds between the drug’s ammine ligands and the carbonyl oxygens on the macrocycle’s portal.^28,29^ This binding mode resulted in steric hindrance at the platinum center, thereby protecting the drug from biological nucleophiles like glutathione or proteins containing accessible thiols and thioethers, e.g., cysteine and methionine residues. Also, the encapsulation of cisplatin inside of CB[7] resulted in substantially reduced release rates of cisplatin in vivo compared to in vitro experiments. The longer circulation times of cisplatin@CB[7] compared to the free drug led to better efficacy based on a pharmacokinetic effect.^29^

Marek and co-workers investigated CB[*n*]s as macrocyclic hosts for potential ruthenium(II)^30^ and paramagnetic ruthenium(III) metallodrugs.^31,32^ Inclusion complexes were formed with specific groups on the ligands of the ruthenium centers, e.g., adamantane moieties. The ruthenium atom was located outside the cavity in all cases.

**Figure 2:**
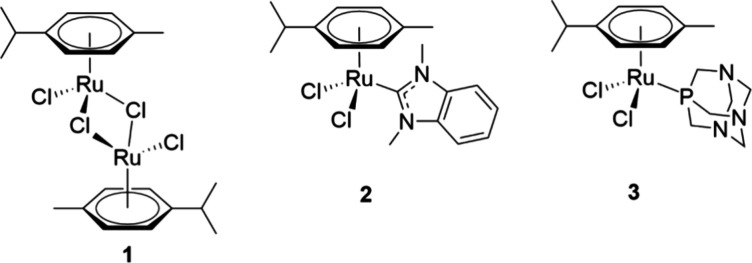
Ruthenium complexes used in this study: ruthenium precursor [Ru^II^(cym)Cl_2_]_2_ (**1**), and ruthenium lead structures [Ru^II^(cym)(dmb)Cl_2_]) (**2**), and [Ru^II^(cym)(pta)Cl_2_] (RAPTA-C, **3**).

In the current study, we investigated the ruthenium(II) arene anticancer lead structures [Ru^II^(cym)(dmb)Cl_2_]^17^ (**2**, cym = η^6^-*p*-cymene, dmb **=** 1,3-dimethylbenzimidazol-2-ylidene) and [Ru^II^(cym)(pta)Cl_2_] (RAPTA-C, **3**, pta **=** 1,3,5-triaza-7-phospha-adamantane)^11,12^ as well as the precursor compound [Ru^II^(cym)Cl_2_]_2_ (**1**, see Figure 2) regarding their ability to form host–guest inclusion complexes with CB[7] and CB[8] to evaluate the possibility of using such molecular containers to mitigate the high reactivity of many metal-based drugs and protect them to some degree from premature exposure to biological nucleophiles. In contrast to the existing literature on the encapsulations of ruthenium complexes, no particular anchoring groups were attached in our case. The analogues CB[7] and CB[8] were chosen based on their suitable cavity size.^33^ In this work, ^1^H-nuclear magnetic resonance spectroscopy (NMR), high-resolution electrospray ionization mass spectrometry (HR-ESI–MS), and theoretical calculations at the density functional theory (DFT) level were employed to analyze binding preference, geometry, and gas phase stability.

## Experimental Section

### Materials

[Ru^II^(cym)Cl_2_]_2_ (**1**; cym = η^6^-*p*-cymene) and all other chemicals and reagents for the synthesis of **2** and **3** were purchased from Sigma-Aldrich. NMR solvents were obtained from Eurisotop. Cucurbit[7]uril (CB[7]) and cucurbit[8]uril (CB[8]) were purchased from Aqdot (Cambridge, UK). All of the reagents were used as received. [Ru^II^(cym)(dmb)Cl_2_]) (**2**; cym = η^6^-*p*-cymene; dmb = 1,3-dimethylbenzimidazol-2-ylidene) and [Ru^II^(cym)(pta)Cl_2_] (**3**, RAPTA-C; cym = η^6^-*p*-cymene; pta = 1,3,5-triaza-7-phospha-adamantane) were prepared as previously published.^17,34^

### NMR Experiments

NMR spectroscopy was performed on a Bruker Avance 4 Neo instrument (^1^H resonance frequency: 400 MHz). MestReNova 14.1.1 was used for data analysis.

Ruthenium complexes **1**–**3** were dissolved at 4 mM in either D_2_O or 150 mM NaCl in D_2_O. To push the equilibrium toward the fully hydrolyzed complexes, they were treated with 1 equiv of AgNO_3_ in D_2_O and left to incubate overnight. The formed solid (AgCl) was then filtered off, and the remaining solution was subjected to NMR measurement.

Samples containing complexes **1**–**3** in a 1:1 ratio with CB[7] and CB[8] were also prepared at 4 mM in either D_2_O or 150 mM NaCl in D_2_O, or in D_2_O after treatment with AgNO_3_ and subsequent filtration.

### MS Experiments

High-resolution electrospray ionization mass spectrometry (HR-ESI–MS) was performed using an Orbitrap Elite mass spectrometer (Thermo Fisher Scientific, Waltham, MA, USA) in positive ion mode.

Ruthenium complexes **1**–**3** were mixed in a 1:1:1 ratio with CB[7] and CB[8] at 1 mM. An aliquot of the corresponding solution was diluted with MeOH/H_2_O (1:1) or ACN/H_2_O (1:1) and subjected to MS analysis immediately, after 24 h, 72 h, and 7 days. Typically, sample solutions were infused at 5 μL/min and ionized in the HESI source with standard conditions (HESI temperature 45 °C, 4 kV spray voltage, capillary temperature 275 °C, and sheath gas flow rate at 5 arbitrary units). Ions of interest were isolated with an isolation width of 7 *m*/*z* and subjected to HCD with stepwise increases of fragmentation energy from 0 to 60 units of NCE (normalized collision energy, dimensionless), using the “scan activation parameters” function of the Tune software provided by Thermo Scientific. This process was repeated at least twice for every ion of interest. Data were then analyzed using the Xcalibur software package (Thermo Scientific). Ion intensities of at least three independent experiments were extracted from the raw files and calculated as mean values plus/minus standard deviation in Microsoft Excel. Breakdown curves were plotted from the precursor ion intensities in correlation to the fragment ion intensities in Microsoft Excel. Theoretical *m*/*z* values were calculated with the Ru^102^ and Cl^35^ isotopes.

### Global Geometry Optimization of Guest@CB[*n*] Systems

The interaction between the host systems CB[7] and CB[8] and the ruthenium guest complexes **2** and **3** have been determined using a basin hopping global minimization strategy.^35^ The starting structures have been generated by inserting the respective guest molecules into the cavity of preoptimized CB[7] and CB[8] molecules. The guest molecules were rotated along the *x*, *y*, and *z* axes from 0° to 180° in increments of 15°. Additionally, a horizontal shift along the *z*-axis of the ruthenium(II) arene complexes from −3.0 to 3.0 Å in increments of 1.0 Å was considered. Individual starting structures with a minimum distance of 1.0 Å between all atoms of the host systems and the guest were generated and optimized. In addition, the structures of isolated CB[7] and CB[8] as well as the guest complexes **2** and **3** have been optimized.

The preoptimization step was carried out employing the semiempirical second-generation extended tight binding method for geometries, frequencies, and nonbonded interactions (GFN2-xTB).^36−38^ The most stable conformations of each combination of guest@CB[*n*] (*n* = 7, 8) based on the resulting interaction energy as well as isolated structures of CB[7] and CB[8] and ruthenium complexes **2** and **3** were further subjected to energy minimization at the Becke 3-parameter Lee–Yang–Parr (B3LYP)^39−41^ level of theory with and without the inclusion of D3 dispersion correction^42−44^ (including Becke–Johnson damping, BJ)^45^ within the resolution of the identity (RI) framework.^46−51^ The def2-SVP^52^ basis set in conjunction with the def2-SVP^47,53^ auxiliary basis set was used for all atoms. For the ruthenium atom, a quasi-relativistic ECP-28-MWB Stuttgart/Dresden pseudopotential effective core potential (ECP) was employed.^54^ The calculations at GFN2-xTB and RIB3LYP levels were executed using DFTB+ (v 22.2)^55^ and TURBOMOLE,^56^ respectively.

The interaction energy *E*_int_ has been determined as the energy difference between the complexes@CB[*n*] (*n* = 7, 8) and the isolated CB[*n*] as well as the guest complex energy via:
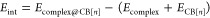
1

## Results and Discussion

### Hydrolysis of Ruthenium Complexes in Aqueous Environment

The ligand exchange reactions of labile ligands are relevant to many metallodrugs. Cisplatin, for instance, retains its chlorido ligands as long it resides in the high Cl^–^ concentration of the bloodstream and undergoes aquation upon entering the environment with low Cl^–^ concentration inside the cell.^57^

**Scheme 1:**
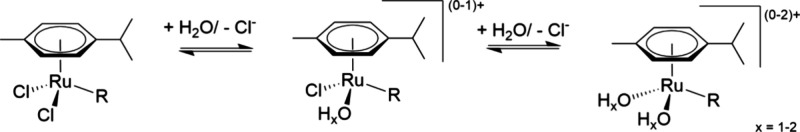
Stepwise Aquation of Ruthenium(II) Arene Complexes

Ruthenium complexes **1**–**3** exchange their chlorido ligands for aqua ligands in aqueous media (see Scheme 1), which results in charged complexes. Depending on its p*K*_a_, the coordinated water molecule may also be present as hydroxido species,^58^ which also influences the overall charge of the ruthenium complexes. In order to monitor the aquation of complexes **1**–**3** and differentiate the various species, ^1^H NMR spectra were recorded in D_2_O 2h after dissolving the complexes. Further spectra were recorded of the species containing both chlorido ligands and the fully aquated species by pushing the equilibrium to either side through the addition of NaCl or AgNO_3_ (see Figures S1–S3). The D_2_O spectra showed a mix of species for all three complexes. The ruthenium dimer **1** was partially hydrolyzed to form monomeric ruthenium arene complexes with different numbers of chlorido and aqua/hydroxido ligands, as evidenced by mass spectrometry (see Table S1). Mass spectra of the aqueous solutions of **1**–**3** showed predominantly singly charged ions of the type [Ru(cym)(dmb/pta)_0–1_Cl]^+^ and some solvent or water adducts (see Table S1). The exact composition of species in solution, however, remains elusive.

### Host–Guest Binding Studies by NMR

It is expected that aquation and the resulting charge of the ruthenium complexes will influence binding with a molecular host. Therefore, ^1^H NMR spectra of 1:1 mixtures of complexes **1**–**3** with either CB[7] or CB[8] were recorded in D_2_O alone, in 150 mM NaCl and in D_2_O after treatment with 1 eq. of AgNO_3_ and subsequent filtration of the precipitated AgCl. In the latter case, CB[*n*] was added after filtration, hence to the fully aquated species. Binding events of the host and guest can be monitored in ^1^H NMR spectra through changes in the chemical shifts of the guest’s protons inside the cavity (upfield) and in the portal region of the CBs (downfield).^59^ Signals are often broadened, which reflects the equilibrium between the guest molecule residing inside and outside of the cavity. In some cases, the exchange kinetics are sufficiently slow on the NMR time scale to observe two separate signals for bound and unbound guests.

**Figure 3:**
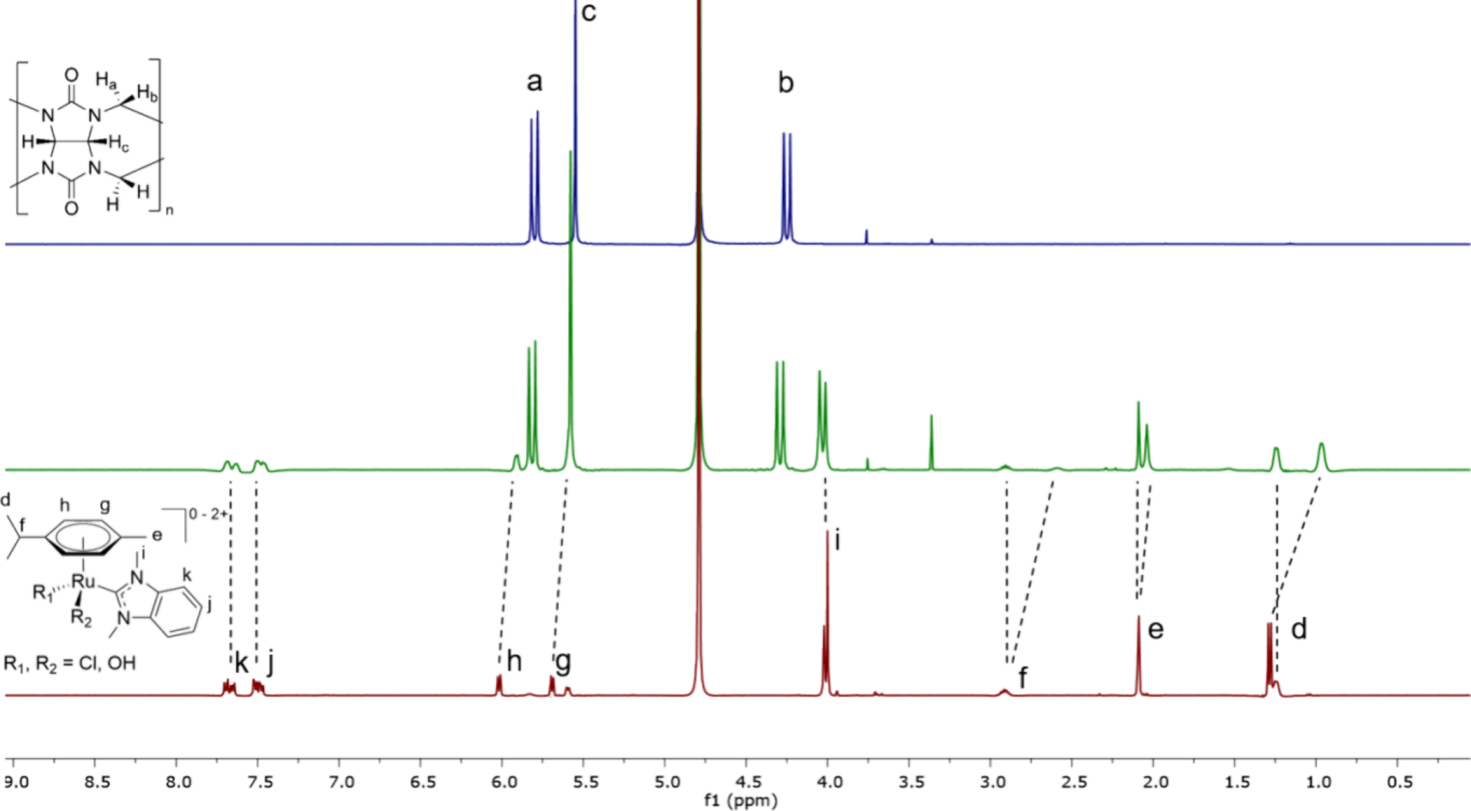
^1^H NMR spectra in D_2_O: (top) 4 mM CB[7], (middle) 1:1 mixture of **2** and CB[7] at 4 mM, and (bottom) **2** at 4 mM. CB[7] was used as received from Aqdot and seemed to contain residual solvent signals from synthesis and purification at 3.76 ppm (dioxane) and 3.36 ppm (methanol).

The spectra of complexes **1**–**3** with CB[7] in pure D_2_O (Figures 3, S4, and S5) show a mixture of species: a portion of the guest signals remain sharp at the same ppm value, indicating no binding between the guest and host, while another portion of protons is shifted and broadened, confirming binding to the host. Since the ruthenium compounds **1**–**3** exist in various aquation states in parallel, this finding suggests different affinities for **1**–**3** depending on their aquation state. Figure 3 shows a 1:1 mixture of complex **2** and CB[7] in D_2_O (middle) in comparison to **2** on its own in D_2_O (bottom) and CB[7] (top). The coexistence of at least two species with different numbers of chlorido and aqua ligands is apparent. One species appears to bind to CB[7] as indicated by broadening and upfield shift of the signals, while the signals for another species remain at the same ppm value, pointing to no interaction with CB[7]. Also, protons a–c of the molecular host are slightly shifted upfield, further confirming a host–guest binding event. Similar spectra were observed for ruthenium complexes **1** and **3** with CB[7] (see Figures S4 and S5).

CB[8] has a very low water solubility on its own (<0.01 mM)^33^ but dissolves better upon binding a suitable guest molecule. Therefore, it was not possible to record an ^1^H NMR spectrum of CB[8] on its own in D_2_O. However, mixing of complexes **1**–**3** with CB[8] in D_2_O resulted in (partial) dissolution of CB[8], which is already evidence for host–guest interactions. In general, the spectra of **1**–**3** with CB[8] after filtration of any remaining solid (Figures S6–S8) showed very broad signals for the ruthenium species, and no unbound species were evident.

**Figure 4:**
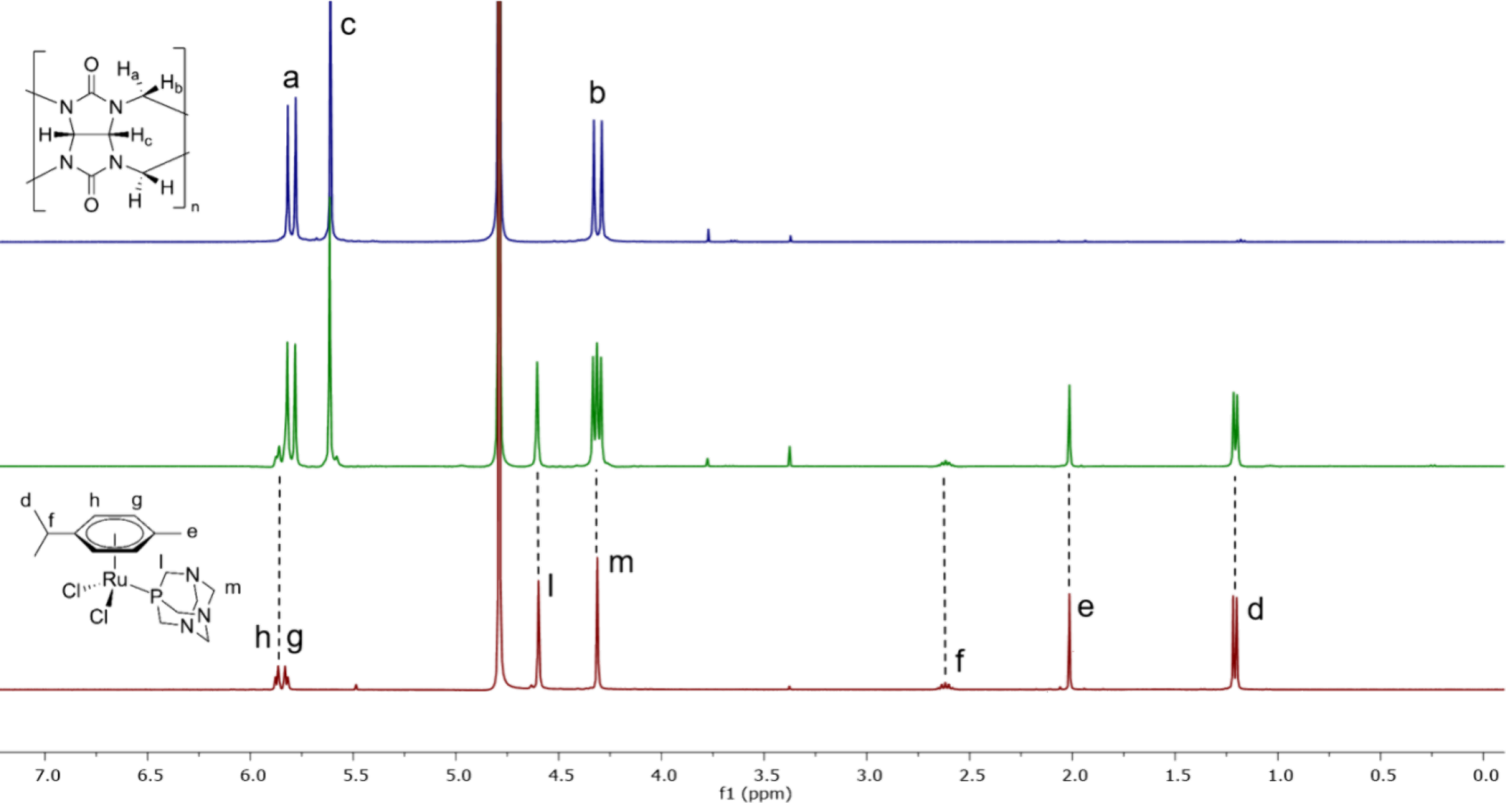
^1^H NMR spectra in 150 mM NaCl: (top) 4 mM CB[7], (middle) 1:1 mixture of **3** and CB[7] at 4 mM, and (bottom) **3** at 4 mM.

To further elucidate the capacity of ruthenium compounds **1**–**3** to bind inside of CB[7/8], additional NMR experiments were performed in 150 mM NaCl. As an example, Figure 4 depicts that RAPTA-C (**3**) does not bind to CB[7] in the presence of 150 mM NaCl as the respective chemical shifts for the protons of **3** remain the same after addition of CB[7]. Also, no binding was observed for **3** and CB[8] since the NMR spectrum of the respective mixture did not contain any CB[8] after filtration (Figure S13). In contrast, shifts and broadening were observed for complexes **1** and **2,** with both CB[7] and CB[8] in 150 mM NaCl, indicating at least partial binding in these cases (Figures S9–S12).

**Figure 5:**
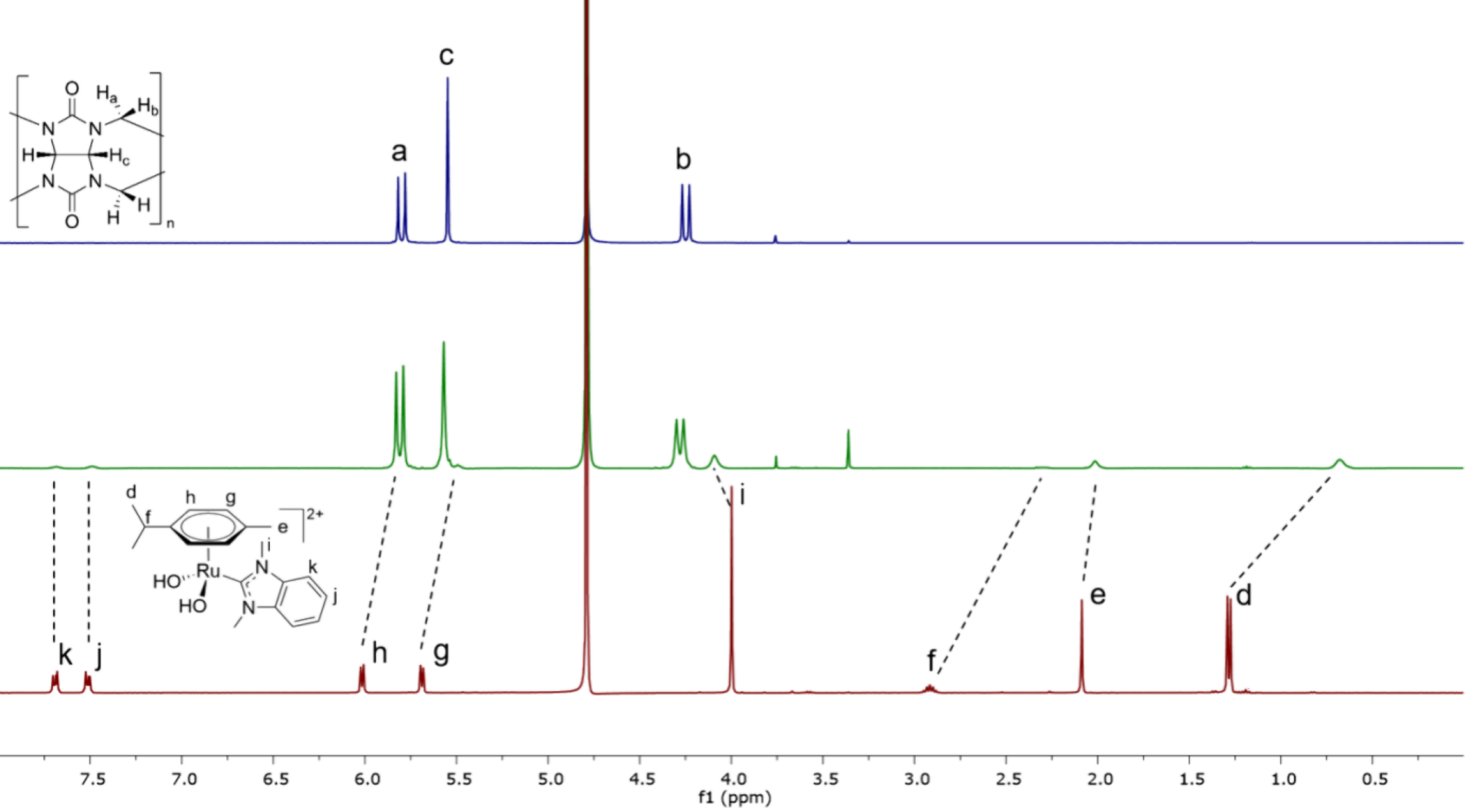
^1^H NMR spectra in D_2_O: (top) 4 mM CB[7], (middle) 1:1 mixture of **2** at 4 mM, after treatment with AgNO_3_, and CB[7] (4 mM), and (bottom) **2** at 4 mM, after treatment with AgNO_3_.

To push the equilibrium toward the aqua complexes, compounds **1**–**3** were mixed with 1 eq. of AgNO_3_, the resulting solid of AgCl was filtered off, and CB[*n*]s were added in a 1:1 ratio to the remaining solution. Figure 5 shows the formation of an inclusion complex of the aquaspecies of **2** with CB[7], as evident from the upfield shift and broadening of signals. The largest shift is observed for the isopropyl group on the arene ligand, indicating that this part of the molecule is immersed the most in the cavity of CB[7]. The protons of the dmb ligand at 7.51 (*k*) and 7.69 ppm (*j*) are broadened but not shifted, and the *N*-methyl protons (*i* at 4.00 ppm) are shifted downfield, which is evidence for the dmb ligand being located close to the portal of CB[7]. The ^1^H NMR spectrum of the same ruthenium complex **2** with CB[8] is less clear with all signals being very broad (see Figure S17), which points to either the whole aqua complex of **2** residing inside the cavity or the existence of two equivalent binding modes where the dmb ligand points inward in one case and the arene ligand in the other case. The charged ruthenium(II) center is expected to form ion dipole interactions with the carbonyl groups on the rims of the CB[n]s.

In the case of ruthenium dimer **1**, inclusion of the arene ligand with the isopropyl group pointing inside the cavities of CB[7] and CB[8] was observed (see Figures S14 and S16). Complex **3** also binds via the arene ligand to CB[8] with the pta ligand being close to the portal region (see Figure S18). However, **3**@CB[7] (Figure S15) displayed downfield shifts for the arene signals and upfield shifts for the protons of the pta ligand, indicating binding through the pta ligand in this case. Hence, different binding geometries are possible for **2** and **3**, and the size of the molecular host determines the preferred binding mode, i.e., which ligand points into the cavity. These observations were further investigated by HR-ESI–MS and computational methods.

### Competition Experiments by MS

In addition to the investigations in solution by NMR spectroscopy, HR-ESI–MS was used to shed further light on the interactions of ruthenium arene complexes **1**–**3** with CB[7] and CB[8]. Gas-phase studies are well-suited to study the intrinsic properties of noncovalent assemblies, as interfering effects of the surrounding environment are eliminated.^60^ Competition experiments were employed to determine binding preferences rooted in the different sizes and geometries of the ligands in **1**–**3**. The respective ruthenium complexes and both CBs were mixed in a 1:1:1 ratio in pure water without any additives and diluted with ACN or MeOH containing 0.1% formic acid to improve spray stability and signal/noise ratio compared to pure water measurements. Labile chlorido or aqua ligands are typically lost upon transfer to the gas phase. Spectra were acquired immediately, after 24 h, 72 h, and 7 days, with no significant changes being observed over this time frame.

**Figure 6:**
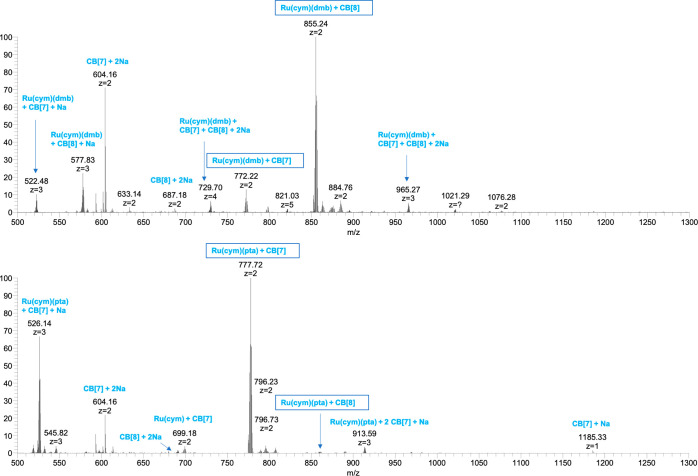
Relevant range of the full HR-ESI mass spectra of a 1:1:1 mixture of complex **2** (top) and complex **3** (bottom) with CB[7] and CB[8].

A noncovalent assembly of the intact ruthenium dimer **1** with any CB[n] was not observed in the gas phase; however, its degradation product formed low abundant inclusion complex ions of the type [Ru(cym) + CB[7]]^2+^ with *m*/*z* 699.18 and to a slightly larger amount [Ru(cym) + CB[8] + ACN]^2+^ with *m*/*z* 802.72 (see Figure S19), indicating a preference of the [Ru(cym)]^2+^ moiety for the larger host. Figure 6 shows that complex **2** clearly favors CB[8] over CB[7] as the relative abundances of the respective ions in the mass spectrum are very different and reflect the ratio of species in solution for compounds with comparable ionizability. In contrast, the **3**@CB[7] ion is roughly 100 times more abundant than the respective host–guest complex with CB[8]. These findings match well with the NMR data, which suggested that the pta ligand on **3** fits the CB[7] cavity better than the cym ligand which is present on all three ruthenium complexes. The dmb ligand, on the other hand, seems to fit nicely into the larger cavity of CB[8].

### Gas Phase Stability of Inclusion Complexes

**Figure 7:**
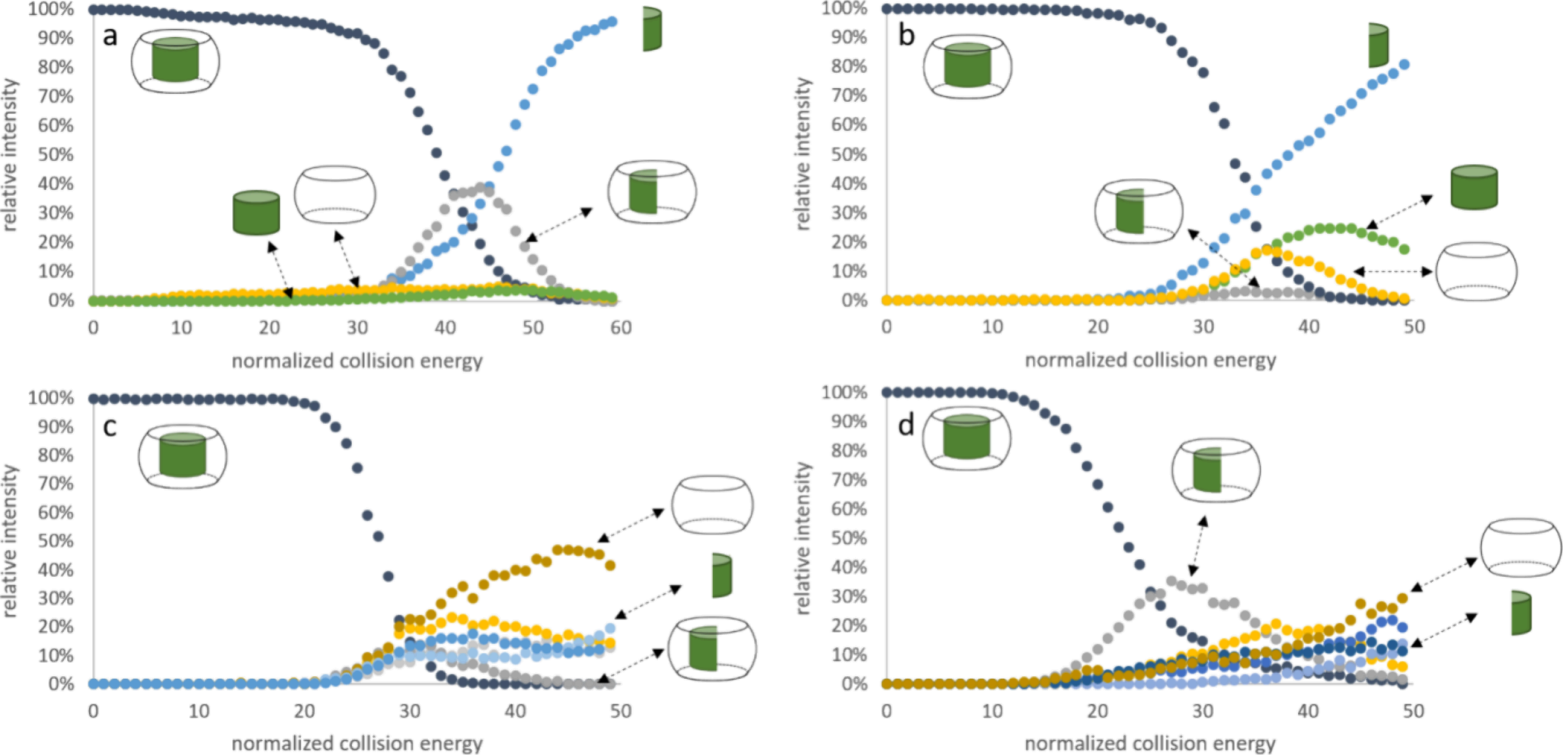
Precursor and fragment ion intensities as a function of normalized collision energy, i.e., ER–MS plots of **2**@CB[7] (a), **2**@CB[8] (b), **3**@CB[7] (c), and **3**@CB[8] (d).

Energy-resolved mass spectrometry (ER–MS) is a tool that allows deeper insights into the noncovalent binding of host–guest systems.^61^ The different fragmentation channels provide information about the geometry of the noncovalent assembly. Also, the relative gas phase stability of a series of similar ions can be measured.^62^ Here, the respective 1:1 inclusion complexes of **2** and **3** in CB[7] and CB[8] were isolated in the gas phase and subjected to increasing amounts of normalized collision energy (NCE). Figure 7 shows clear differences in the loss of precursor ion intensities as well as the emerging fragments for various combinations of ruthenium compounds and CB[n]s. All fragment ions are listed in Table S2 in the Supporting Information. The gas phase release of an intact guest ion [Ru(cym)(dmb) – H]^+^ with *m*/*z* 381 was only observed for **2**@CB[8], while in all other cases, the ruthenium compounds disassembled, and a part of it remained bound to the host. For **2**@CB[7], the dmb ligand was cleaved off and became the highest abundant ion with a *m*/*z* of 147.09 for [dmb + H]^+^, followed by the remaining [CB[7] + Ru(cym)]^2+^ with a *m*/*z* of 699.18. No other high abundant fragments were observed in this case. Complex **3** resulted in more fragmentation channels resulting from both cleavage of cym and pta ligands as well as disassembly of the pta ligand. For **3**@CB[7], both fragment ions [CB[7] + Ru(cym)]^2+^ with *m*/*z* 699.18 and [CB[7] + Ru(pta)]^2+^ with *m*/*z* 710.66 emerged with similar intensities, pointing toward two different binding geometries. In the experiments of **3** with CB[8], however, only [CB[8] + Ru(pta)]^2+^ with *m*/*z* 793.68 was observed.

The NCE values at which the precursor ion intensities drop are different for all four host–guest systems and follow the order **2**@CB[7] > **2**@CB[8] > **3**@CB[7] > **3**@CB[8]. However, these gas phase stability values do not reflect the observed binding preferences as observed in the competition experiments. These findings might point toward the observation of both kinetically favored and thermodynamically favored host–guest complexes.

### Structures and Energetics of Guest@CB[*n*] Systems

In order to obtain further insights into the host–guest interactions at the molecular level, interactions between compounds **2** and **3** and CB[*n*] (*n* = 7, 8) have been determined using a basin hopping global minimization approach. A series of individual geometry optimizations have been carried out using the GFN2-xTB method by placing **2** and **3** inside the cavities of CB[7] and CB[8]. The guest molecules were rotated along the *x*, *y*, and *z* directions and shifted in *z* directions to generate a variety of possible geometries showing a minimum distance of 1 Å between the atoms of the guest and the host system. This resulted in 723 to 5055 individual starting structures per guest@host complex that were subjected to geometry optimization.

The calculated interaction energy *E*_int_ obtained for different guest@CB[*n*] (*n* = 7, 8) complexes at the GFN2-xTB level of theory sorted in decreasing order ranges from −2654.5 to −3264.1 kJ mol^–1^ (see Figure S20). The interaction energy reflects the stability of the host–guest complex and is determined as the total energy difference between the encapsulated system and the isolated guest and host molecules.^63^ In all cases, the encapsulation of **2** and **3**@CB[*n*] is energetically favorable, as indicated by the respective negative interaction energies.^63,64^ In order to further evaluate the orientational preference of the arene ligand of complexes **2** and **3** within the cavity of CB[*n*], the optimized structures of the different guest@CB[*n*] systems showing the lowest *E*_int_ value obtained at the GFN2-xTB level were then subjected to energy minimization at the RIB3LYP and RIB3LYP-D3 levels of theory. Both variants with the arene ligand inside and outside the CB[*n*] structure were considered.

**Figure 8:**
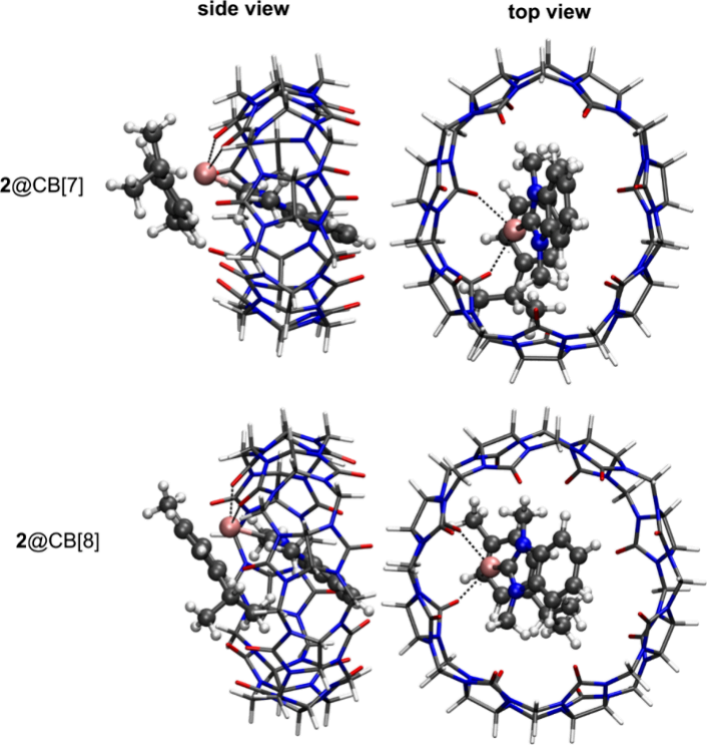
Optimized structures of **2**@CB[7] and **2**@CB[8] showing the lowest interaction energy *E*_int_ at RIB3LYP-D3 level of theory. The arene ligand of complex **2** is located outside of the CB host system.

**Figure 9:**
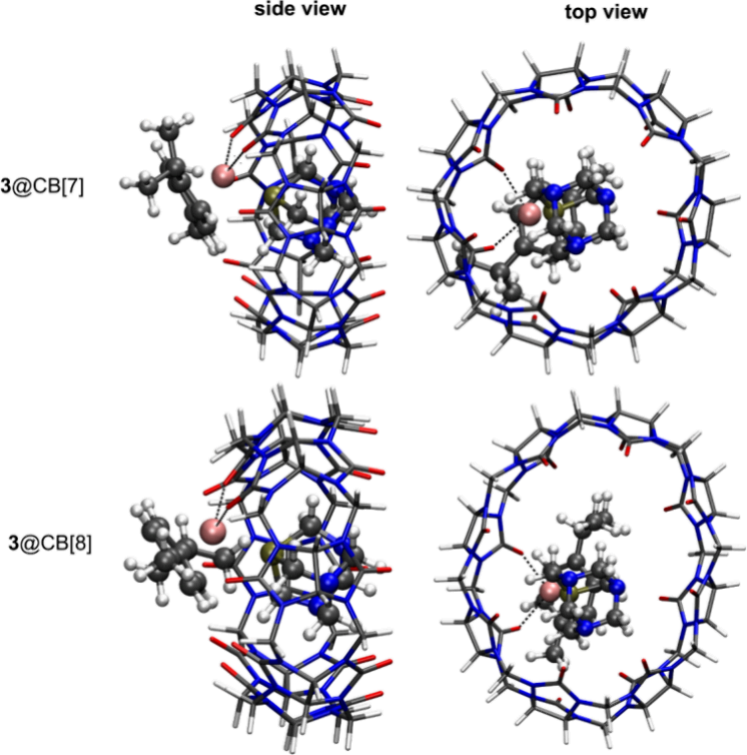
Optimized structures of **3**@CB[7] and **3**@CB[8] showing the lowest interaction energy *E*_int_ at RIB3LYP-D3 level of theory. The arene ligand of complex **3** is located outside the CB host system.

The optimized geometries of compounds **2** and **3** bound to the CB[*n*] host systems obtained at the RIB3LYP-D3 level of theory are, respectively, shown in Figures 8 and 9, and the respective *E*_int_ values obtained via energy minimization with and without dispersion correction are listed in Table 1. The inclusion of dispersion correction results in a stabilization of the guest@host systems of approximately 30 kJ mol^–1^. This finding is in agreement with the report by Venkataramanan and Suvitha,^65^ highlighting that dispersive effects are essential when investigating inclusion compounds of cucurbiturils. When considering compound **3,** the negative values of Δ*E*_int_ of −52.9 and −81.3 kJ mol^–1^ obtained at RIB3LYP and RIB3LYP-D3 levels of theory provide direct evidence that complex **3** binds predominantly to the CB[7] host molecule. This finding is consistent with the results of the MS competition experiments as well as ^1^H NMR data showing that the pta ligand of complex **3** fits the cavity of CB[7] better than the cym ligand. CB[7] has a hydrophobic inner cavity,^66^ while the pta ligand is considered as a bulky neutral ligand,^67^ which makes it more favorable to reside inside the cavity of CB[*n*]. The inclusion of the D3 dispersion correction stabilizes Δ*E*_int_ obtained at the RIB3LYP and RIB3LYP-D3 levels of theory by approximately −28.4 kJ mol^–1^, again highlighting its importance. The ruthenium(II) center of complex **3** binds with two oxygen atoms of carbonyl groups of CB[7] at 2.25 and 2.27 Å.

**Table 1:** Calculated Interaction Energy *E*_int_ in kJ mol^–1^ Obtained for the Most Stable Host–Guest Compounds of **2** and **3** embedded in CB[*n*] (*n* = 7,8) at RIB3LYP and RIB3LYP-D3 Levels of Theory, Respectively^a^

guest	host	*E*_int_	
		RIB3LYP	RIB3LYP-D3
complex **2**	CB[7]	–2153.53	–2405.04
	CB[8]	–2154.71	–2374.81
	Δ*E*_int_	+1.18	–30.23
complex **3**	CB[7]	–2238.08	–2489.29
	CB[8]	–2185.23	–2408.02
	Δ*E*_int_	–52.85	–81.27

a The arene ligand of complexes **2** and **3** is located outside the cavity of the CB host system, as depicted in Figures 8 and 9, respectively.

The optimized geometries of **3**@CB[*n*] at RIB3LYP-D3 level of theory with the arene ligand located inside the cavity of CB[*n*], and the respective *E*_int_ are shown in Figure S22 and Table S3, respectively. Complex **3** prefers binding to CB[7] with the arene ligand located outside rather than inside the cavity of the CB[*n*] with *E*_int_ values of −2489.3 and −2402.5 kJ mol^–1^ at the RIB3LYP-D3 level of theory. In the case of complex **3**@CB[7], it is certainly more favorable for the arene ligand to be located outside the cavity of CB[*n*] with the difference of *E*_int_ of two binding modes being approximately −86.8 kJ mol^–1^. This finding is consistent with ^1^H NMR data, indicating that for **3**@CB[7], the arene ligand is most likely located outside the cavity.

In contrast, a less clear picture is obtained when comparing the *E*_int_ values for complex **2** embedded in the CB[*n*] host. In case of the RIB3LYP-D3 level of theory, the **2**@CB[7] system is favored compared to its **2**@CB[8] counterpart but only by approximately −30.2 kJ mol^–1^. However, in absence of dispersion correction, the energy difference is only +1.2 kJ mol^–1^, implying that both systems have similar interaction energies. The binding of complex **2** into the CB[7] cavity is again stabilized by the ruthenium atom binding to two oxygen atoms of carbonyl groups in the rim of CB[7] at 2.27 and 2.31 Å.

In order to investigate the position preference of the arene ligand@CB[*n*], the optimized geometries and the respective *E*_int_ of complex **2**@CB[*n*] with the arene ligand located inside the cavity of CB[*n*] at the RIB3LYP and RIB3LYP-D3 levels of theory are prepared in Figure S21 and Table S3. The position of the arene ligand is preferentially outside rather than inside the cavity of the CB[*n*] with a Δ*E*_int_ value determined as +10.0 (CB[7]) and +43.4 kJ mol^–1^ (CB[8]) at the RIB3LYP-D3 level of theory. This finding aligns with a previous study by Sojka et al. of RuC-1@CB[6] with the ruthenium caps located outside the CB[6] carrier, thus making them prone to chloride ligand exchange.^30^ Based on these findings, the interaction of complex **2** with the CB[*n*] host system is not as clear as in the case of complex **3** discussed above. Considering the comparably small difference in energy, a competition between CB[7] and CB[8] as well as in terms of the guest orientation in **2**@CB[7] can be expected. This finding aligns well with the experimental data, in particular the ambiguous picture obtained from MS competition experiments and ER–MS data pinpointing to the observation of both, kinetic and thermodynamic inclusion complex formation.

In order to analyze the similarity of optimized structures, root-mean-square deviations (RMSDs) have been determined for the most stable host–guest complex of compounds **2** and **3** embedded in CB[*n*] (*n* = 7, 8) at the GFN2-xTB level in comparison to respective minimum structure reoptimized at RIB3LYP and RIB3LYP-D3 levels of theory (see Table S5). A small deviation is observed in the RMSDs of the optimized structure of **2**@CB[7] at GFN2-xTB in comparison to the RIB3LYP and RIB3LYP-D3 levels of theory, being 0.20 and 0.21 Å, respectively. Similarly, the RMSD of the optimized geometry of **3**@CB[7] at GFN2-xTB in comparison to the RIB3LYP and RIB3LYP-D3 levels of theory are 0.31 and 0.18 Å, respectively. In all cases, smaller RMSD values are observed when comparing the optimized structures of the complex 3@CB[*n*] at the GFN2-xTB level with RIB3LYP-D3 as compared to RIB3LYP. These values indicate that the optimized geometry of **3**@CB[*n*] in GFN2-xTB and RIB3LYP-D3 are not significantly different. The findings support the systematic configurational probing outlined above to first employ a suitable semiempirical method to estimate whether energetically favorable inclusion complexes between CB[*n*] and complexes **2** and **3** can be formed before using the higher level of theory for a final optimization step.

## Conclusions

CB[*n*]s are biocompatible host molecules able to encapsulate small-molecule drugs noncovalently with high affinity, thereby improving solubility and/or providing protection from degradation. The current study sheds light on the suitability of CB[*n*]s as molecular containers for ruthenium(II) arene complexes, with potential applications as anticancer agents. By combining ^1^H NMR, HR-ESI-MS, and quantum chemical calculations, the binding properties of three different ruthenium(II) arene complexes with CB[7] and CB[8] were investigated in detail. Charged metal complexes resulting from the exchange of labile chlorido ligands for aqua ligands exhibited higher binding affinity than their neutral chlorido counterparts, as observed by ^1^H NMR. Competition experiments by HR-ESI–MS revealed that the NHC complex **2** preferentially binds to CB[8], with both the cym and the dmb ligands being immersed in the cavity, probably via two equally stable binding geometries. Complex **3** in contrast exhibited a clear preference for CB[7] with the pta ligand inside and the cym outside the cavity. In all cases, the ruthenium(II) center was located close to the portal interacting with the associated carbonyl groups.

The data obtained from the quantum chemical calculations of the different guest@CB[*n*] systems align very well with the experimental results. In the case of compound **3,** a clear preference for the CB[7] host has been observed based on the comparison of the associated interaction energies. In the most stable configuration, the arene ligand is located outside of the CB[7] cavity. In contrast, the smaller differences in the interaction energies obtained in the case of compound **2** imply a competition between **2**@CB[7] and **2**@CB[8] as well as in terms of the orientation of complex **2** in the CB[7] host. In addition, the comparison of the RMSD values of the optimized structures has shown very good agreement between semiempirical GFN2-xTB data and the high level RIB3LYP-D3 results.

These findings present the first example of direct binding of a ruthenium complex to CB[*n*] through interactions of the carbonyl groups with the metal ion and immersion of one of the larger ligands into the cavity of CB[*n*]. This opens the possibility to develop anticancer metallodrugs encapsulated in CB[*n*]s to mitigate the high reactivity of many metal complexes and protect them to some degree from premature exposure to biological nucleophiles.

## Supporting Information Available

^1^H NMR spectra for all compounds and host guest complexes in all solutions, list of identified ions from MS experiments, theoretical calculations for *E*_int_, and possible binding conformations of the host–guest-complexes (PDF)
