# Supplementary material for: Host–Guest Interactions of Ruthenium(II) Arene Complexes with Cucurbit[7/8]uril
Source: Inorg Chem. 2024 Jul 17;63(30):14021–31. doi: 10.1021/acs.inorgchem.4c01755 (PMC11289748; doi:10.1021/acs.inorgchem.4c01755)
Supplement: Supplementary file 1 — ic4c01755_si_001.pdf [file ic4c01755_si_001.pdf]

SUPPORTING INFORMATION

Host-Guest Interactions of Ruthenium(II) Arene Complexes with Cucurbit[7/8]uril

Elisa Roth,[1#] Risnita Vicky Listyarini,[2,3#] Thomas S. Hofer,[2] Monika Cziferszky*[1]

[1] Institute for Pharmacy, Pharmaceutical Chemistry, Department of Chemistry and Pharmacy, University of Innsbruck, Innrain 80/82, A-6020 Innsbruck, Austria

[2]Institute of General, Inorganic and Theoretical Chemistry, University of Innsbruck, Center for Chemistry and Biomedicine Innrain 80-82, A-6020 Innsbruck, Austria

[3]Chemistry Education Study Program, Sanata Dharma University, Yogyakarta 55282, Indonesia

[#] these authors contributed equally

monika.cziferszky@uibk.ac.at

NMR spectra of 1-3 in aqueous environment 2

Binding studies by NMR 4

^{1}H-NMR spectra in D_2O 4

^{1}H-NMR spectra in 150 mM NaCl 6

^{1}H-NMR spectra in D_2O after treatment with $AgNO_3$ 8

Mass spectrometry 10

Competition experiments 10

Gas phase stability by ER-MS 12

Theoretical calculations 13

NMR spectra of 1-3 in aqueous environment

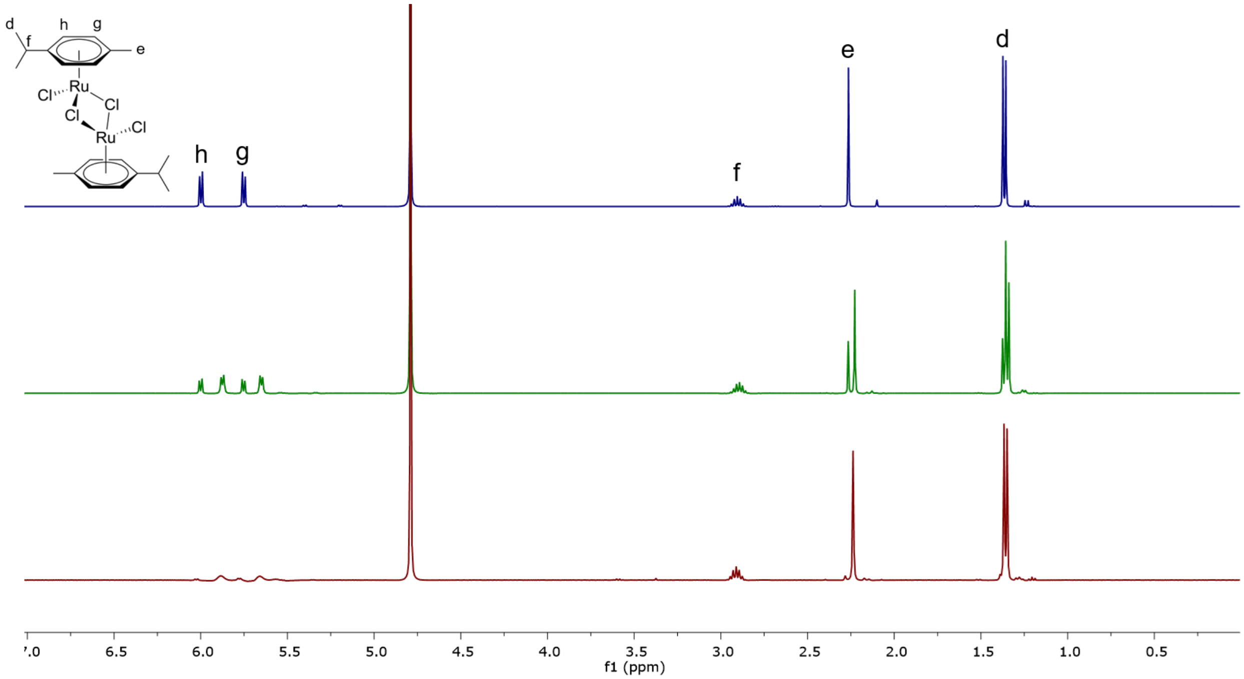

*Figure S 1: ^{1}H-NMR spectra of **1** in 150 mM NaCl in D_2O (top), in pure D_2O 2h after dissolving (middle), and in D_2O after addition of $AgNO_3$ and filtration of the solid (bottom).*

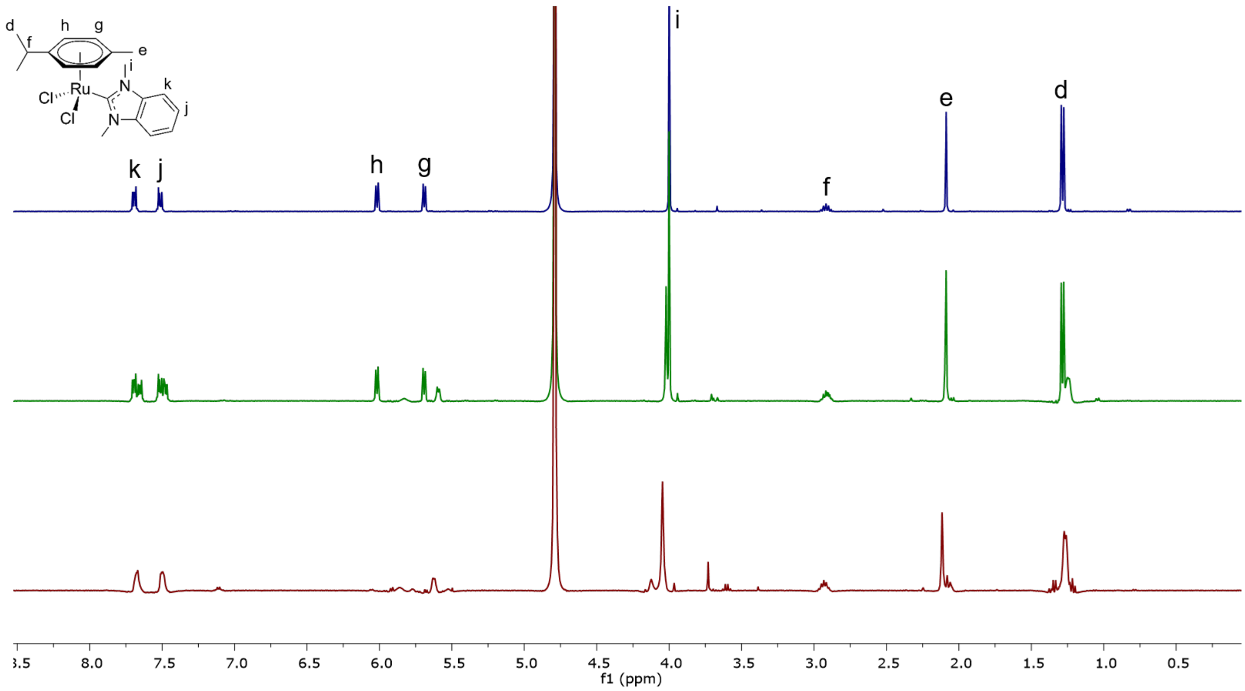

*Figure S 2: ^{1}H-NMR spectra of **2** in 150 mM NaCl in D_2O (top), in pure D_2O 2h after dissolving (middle), and in D_2O after addition of $AgNO_3$ and filtration of the solid (bottom).*

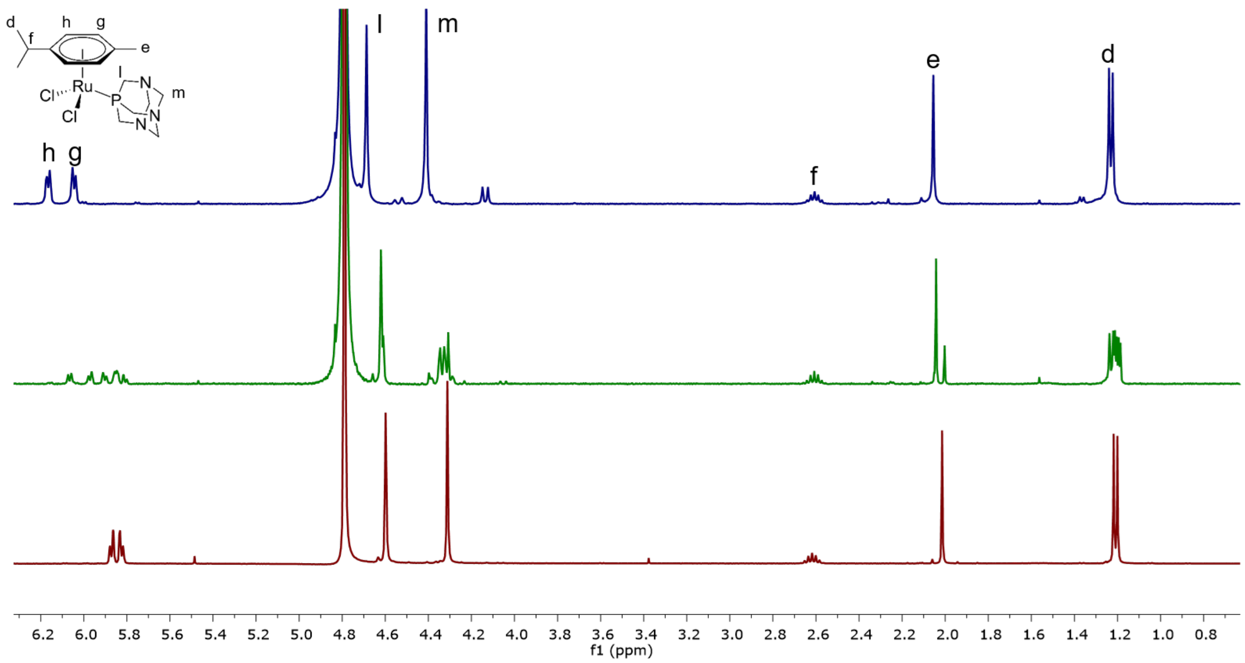

*Figure S 3: ^{1}H-NMR spectra of **3** in 150 mM NaCl in D_2O (top), in pure D_2O 2h after dissolving (middle), and in D_2O after addition of $AgNO_3$ and filtration of the solid (bottom).*

Binding studies by NMR

All ^{1}H-NMR spectra were recorded at 4 mM concentration of the respective compound(s).

^{1}H-NMR spectra in D_2O

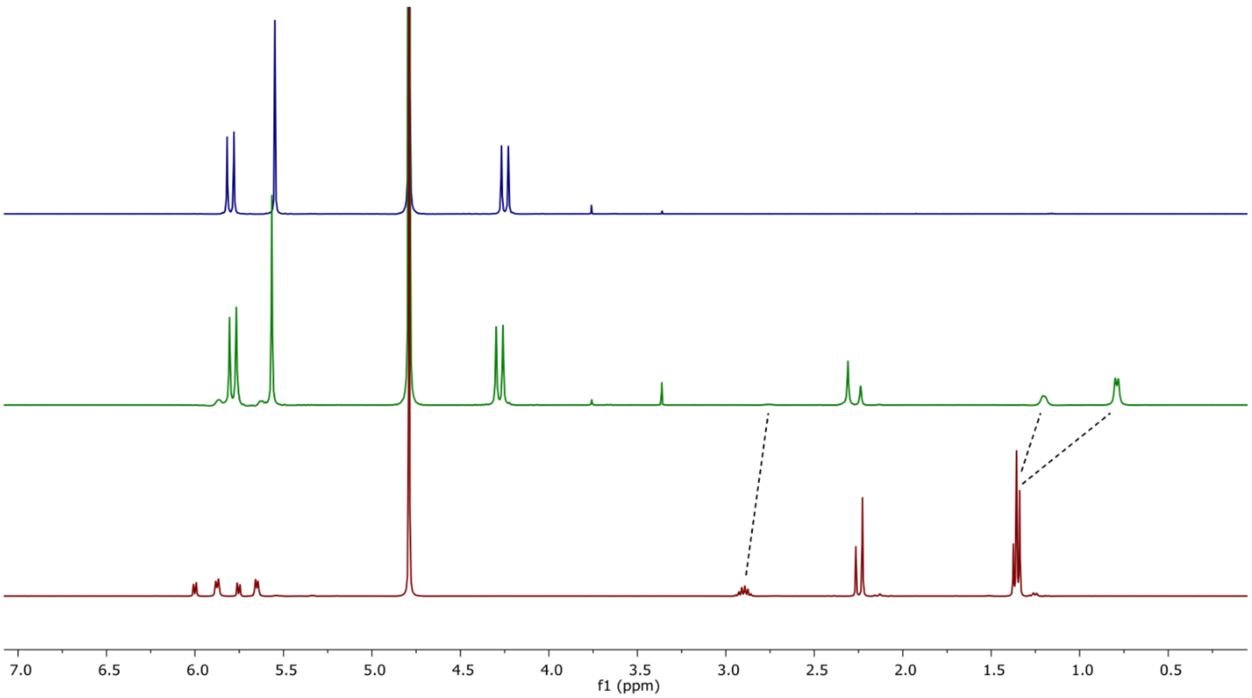

*Figure S 4: ^{1}H-NMR spectra in D_2O of CB[7] (top), a 1:1 mixture of **1** and CB[7] (middle) and **1** (bottom).*

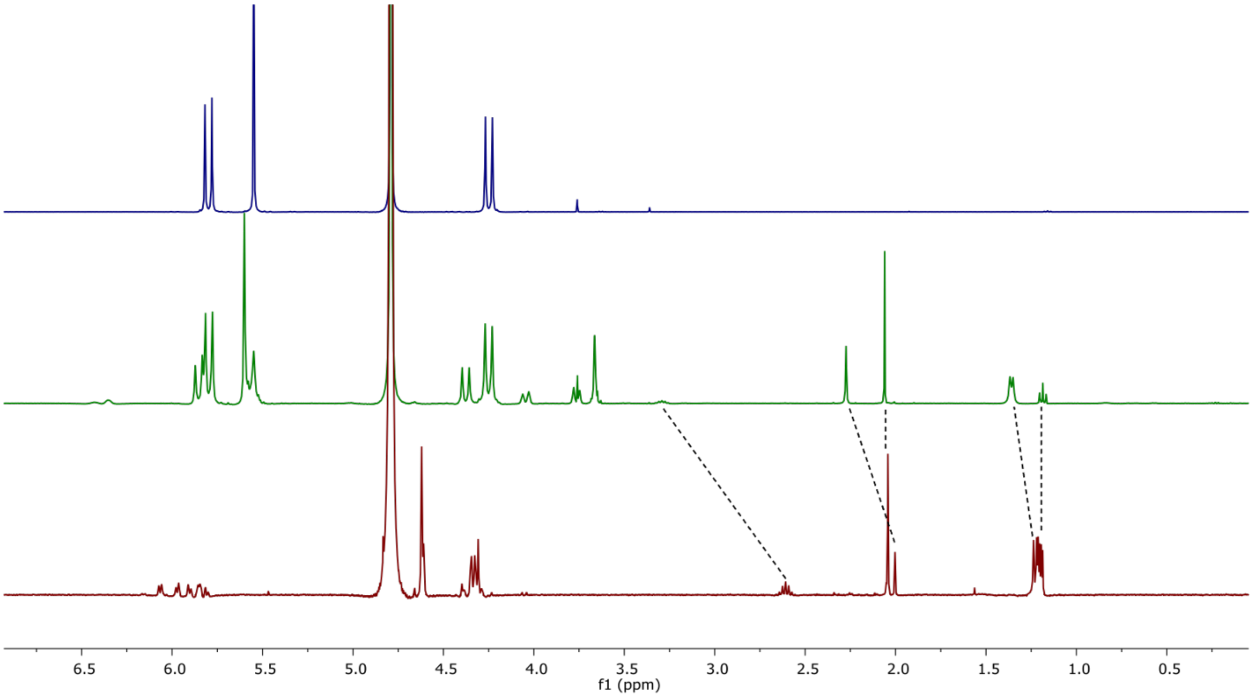

*Figure S 5: ^{1}H-NMR spectra in D_2O of CB[7] (top), a 1:1 mixture of **3** and CB[7] (middle) and **3** (bottom).*

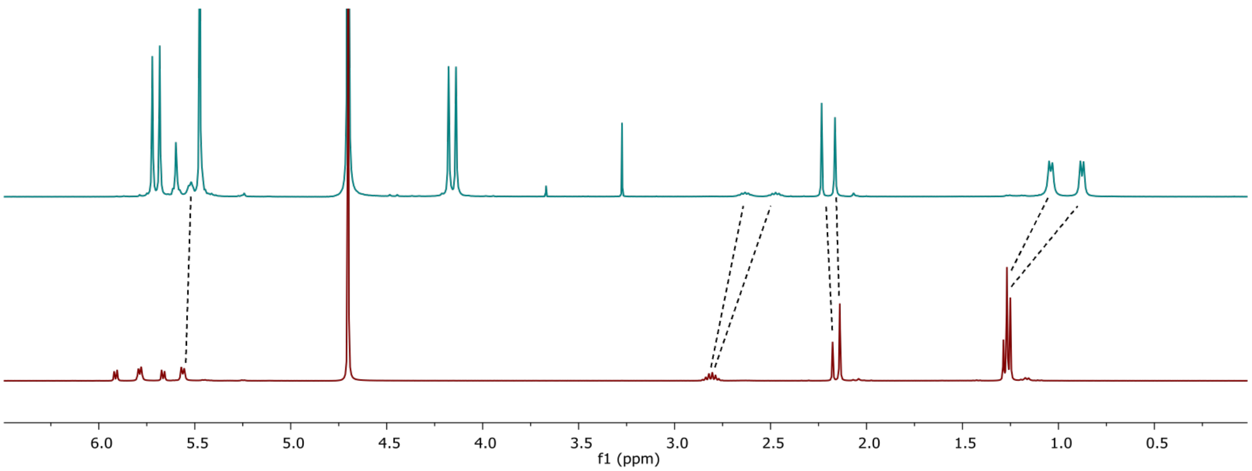

*Figure S 6: ^{1}H-NMR spectra in D_2O of a 1:1 mixture of **1** and CB[8] (top) and **1** (bottom).*

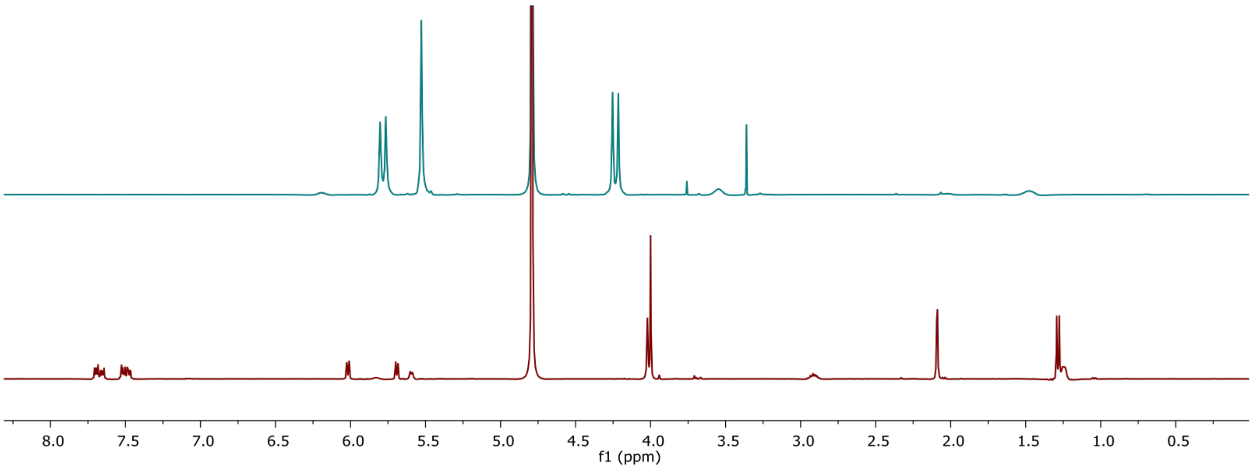

*Figure S 7: ^{1}H-NMR spectra in D_2O of a 1:1 mixture of **2** and CB[8] (top) and **2** (bottom).*

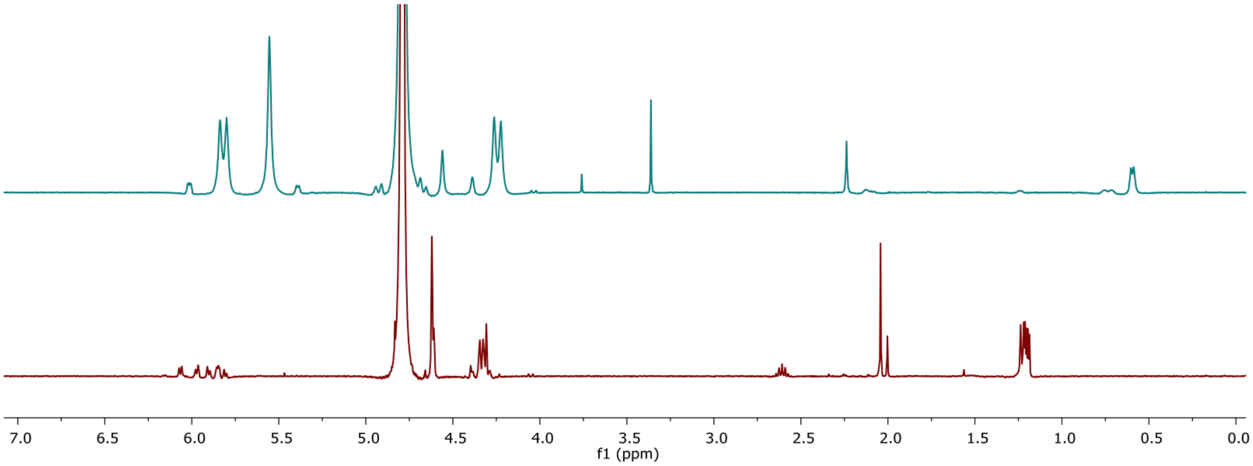

*Figure S 8: ^{1}H-NMR spectra in D_2O of a 1:1 mixture of **3** and CB[8] (top) and **3** (bottom).*

^{1}H-NMR spectra in 150 mM NaCl

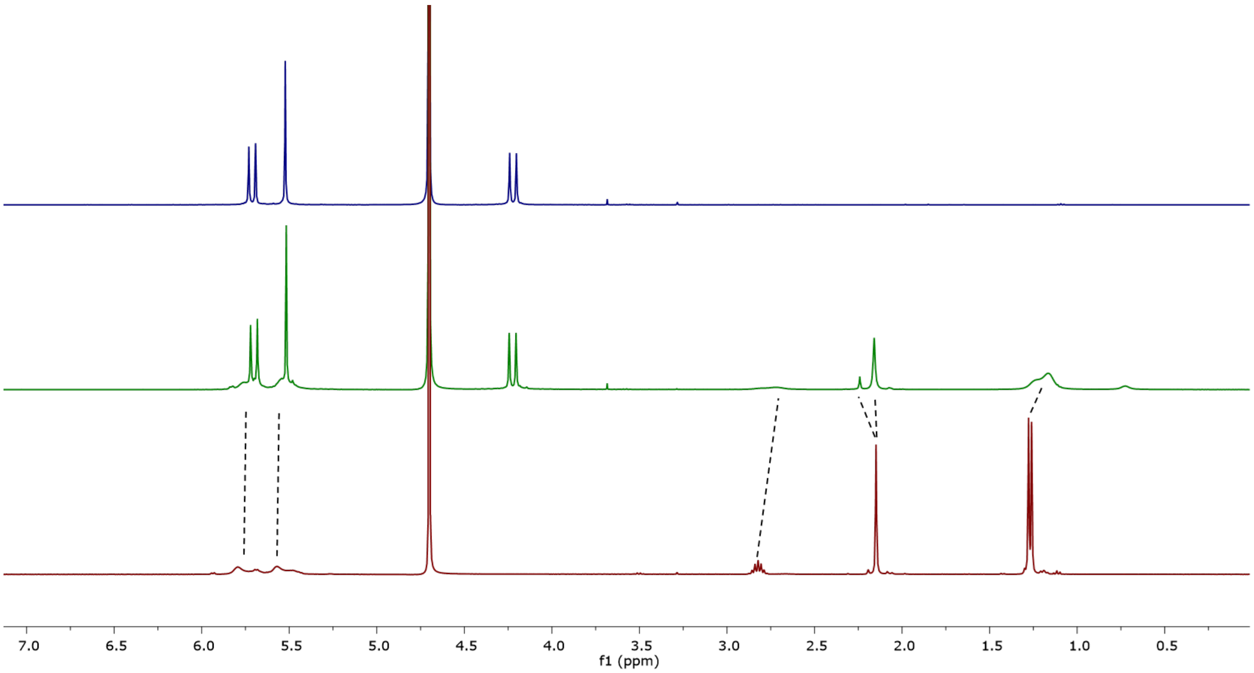

Figure S 9: ^{1}H-NMR spectra in 150 mM NaCl of CB[7] (top), a 1:1 mixture of ***1*** *and CB[7] (middle) and* ***1*** *(bottom).*

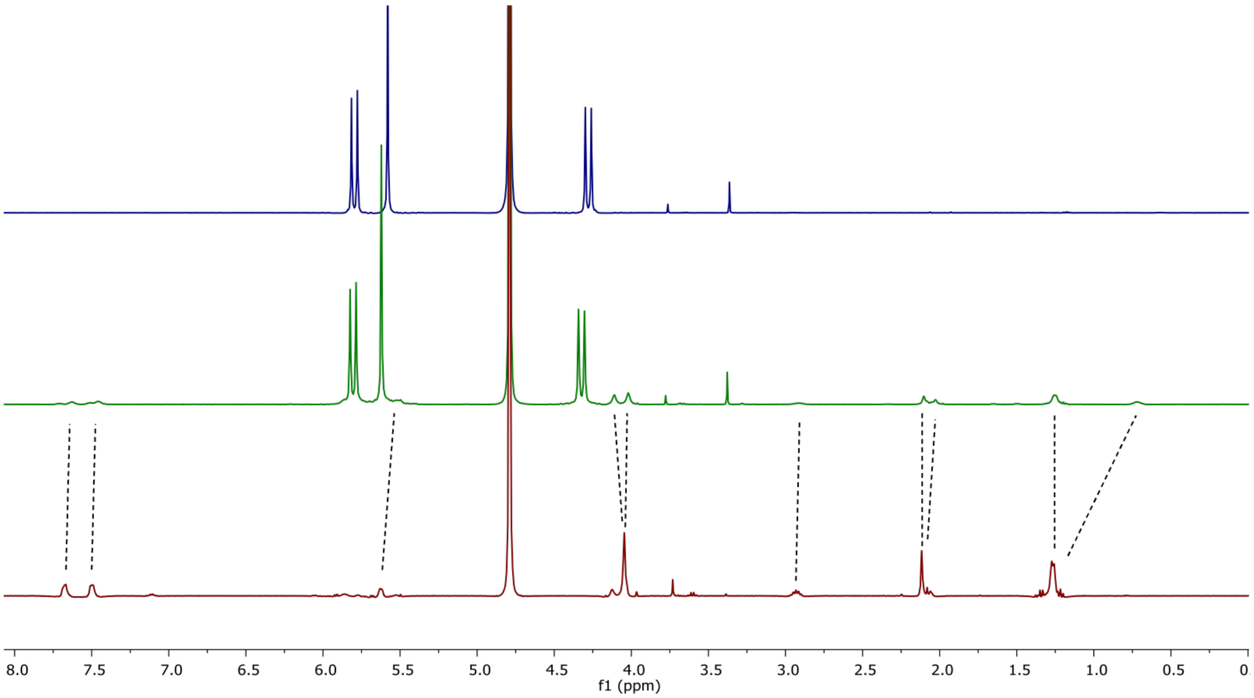

Figure S 10: ^{1}H-NMR spectra in 150 mM NaCl of CB[7] (top), a 1:1 mixture of ***2*** *and CB[7] (middle) and* ***2*** *(bottom).*

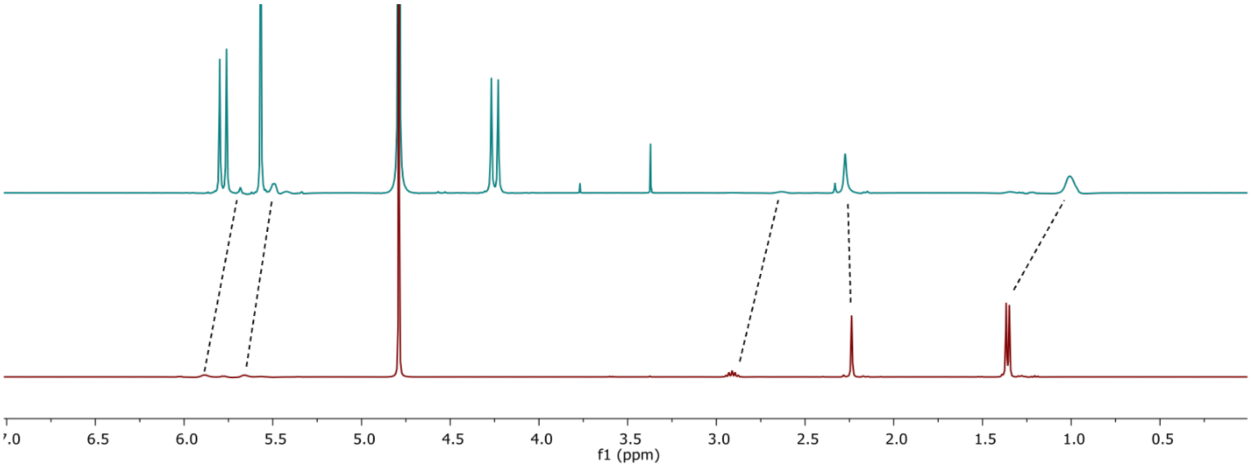

*Figure S 11: ^{1}H-NMR spectra in 150 mM NaCl of a 1:1 mixture of **1** and CB[8] (top) and **1** (bottom).*

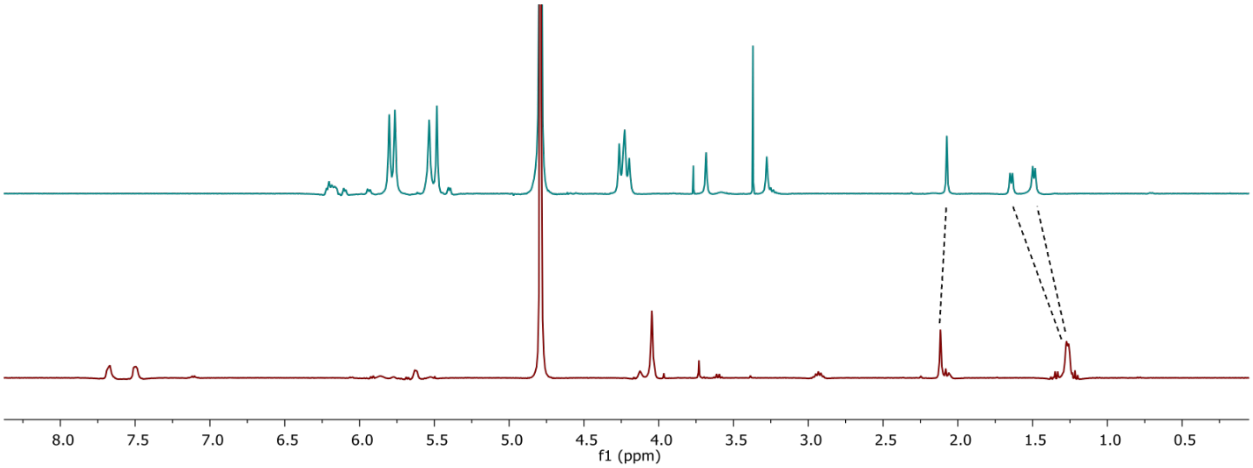

*Figure S 12: ^{1}H-NMR spectra in 150 mM NaCl of a 1:1 mixture of **2** and CB[8] (top) and **2** (bottom).*

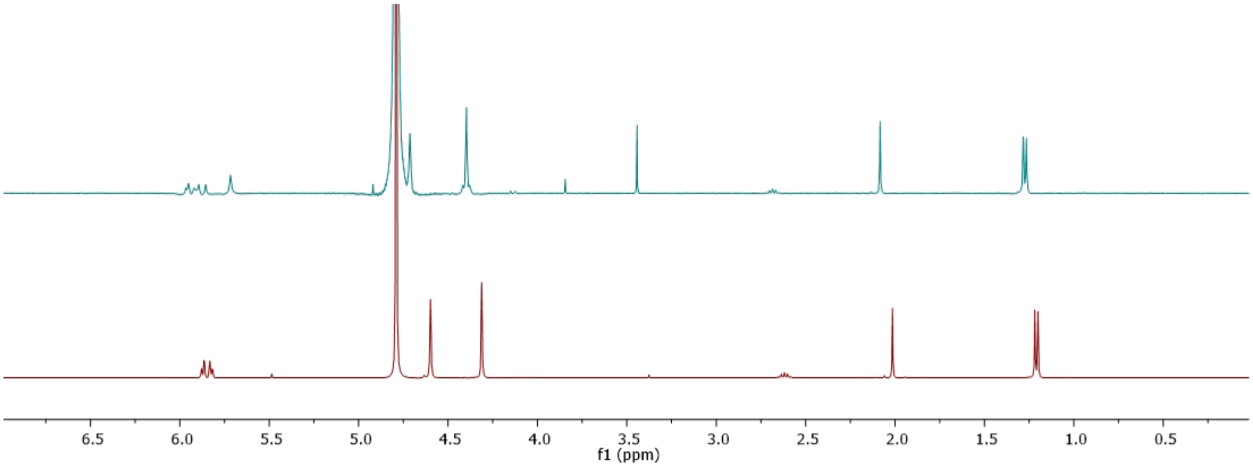

*Figure S 13: ^{1}H-NMR spectra in 150 mM NaCl of a 1:1 mixture of **3** and CB[8] after filtration (top) and **3** (bottom). CB[8] was insoluble in this solution.*

^{1}H-NMR spectra in D_2O after treatment with $AgNO_3$

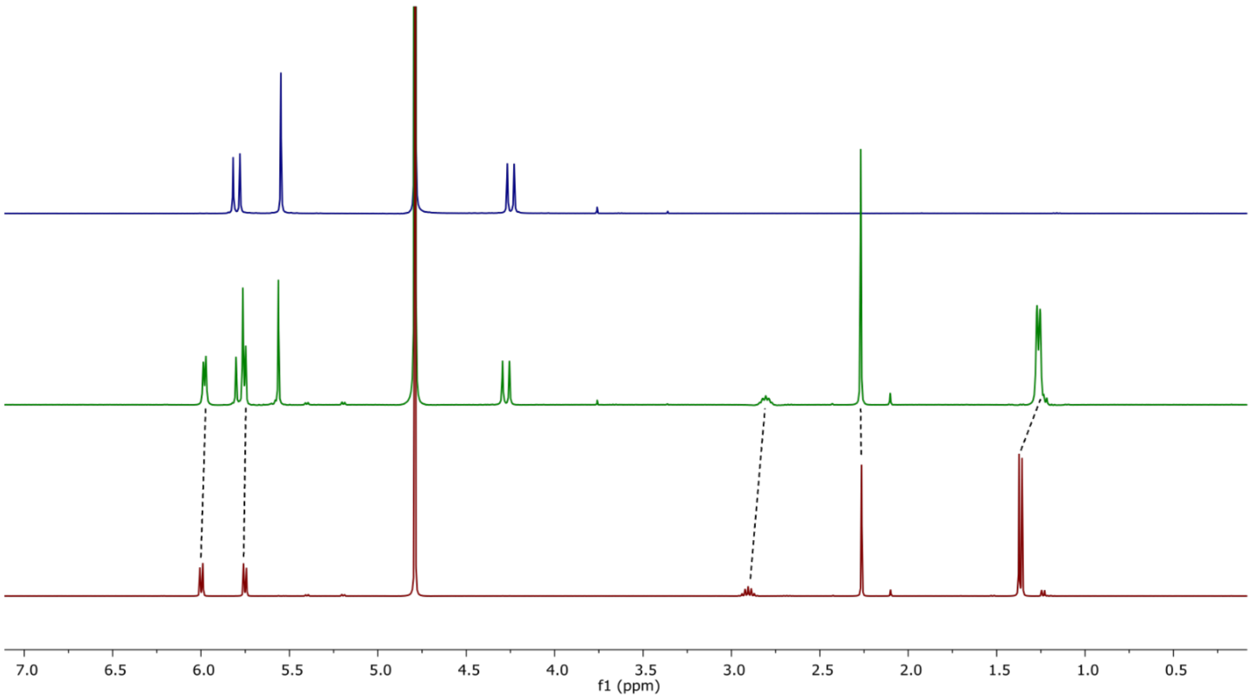

Figure S 14: ^{1}H-NMR spectra in D_2O of CB[7] (top), a 1:1 mixture of ***1*** *after treatment with $AgNO_3$ and CB[7] (middle) and* ***1*** *after treatment with $AgNO_3$ (bottom).*

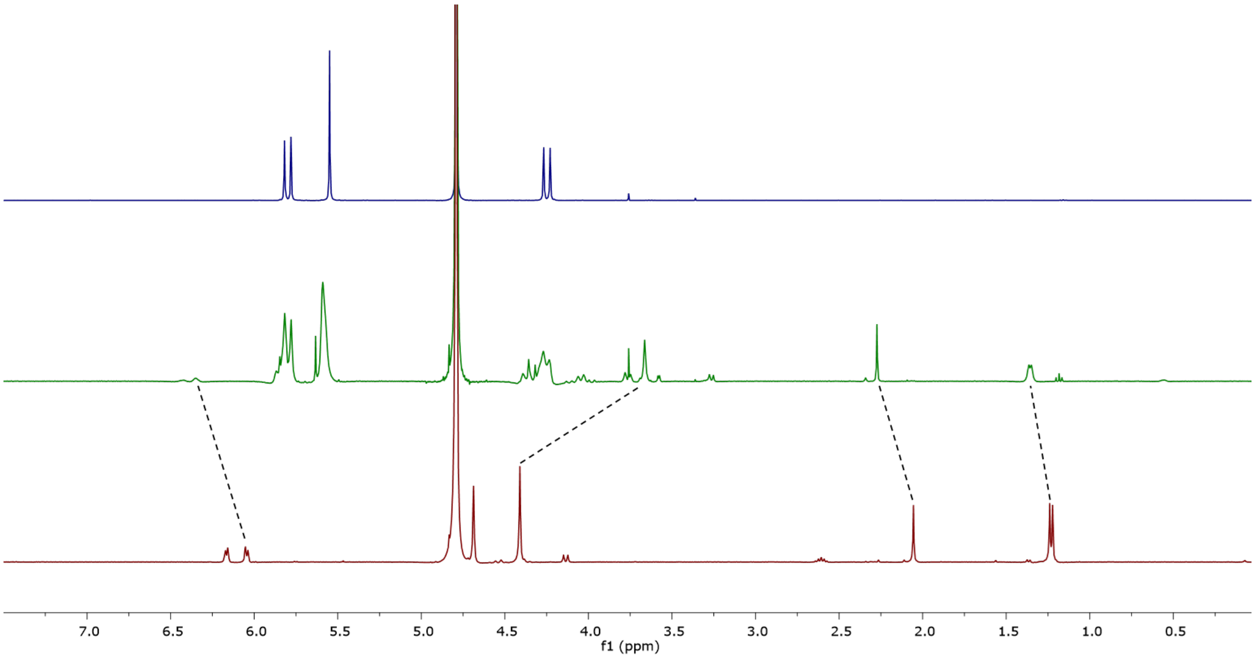

Figure S 15: ^{1}H-NMR spectra in D_2O of CB[7] (top), a 1:1 mixture of ***3*** *after treatment with $AgNO_3$ and CB[7] (middle) and* ***3*** *after treatment with $AgNO_3$ (bottom).*

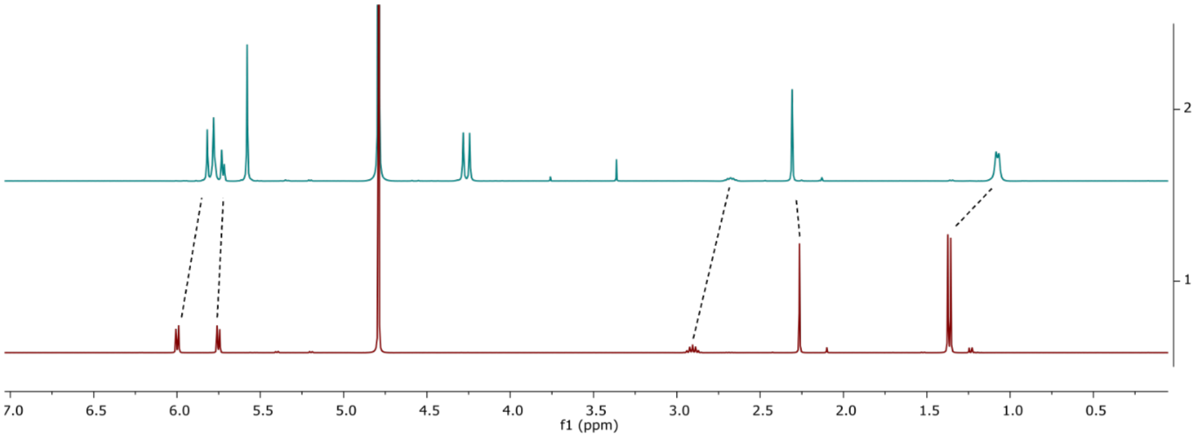

*Figure S 16: ^{1}H-NMR spectra in D_2O of **1** after treatment with $AgNO_3$ and CB[8] (top) and **1** after treatment with $AgNO_3$ (bottom).*

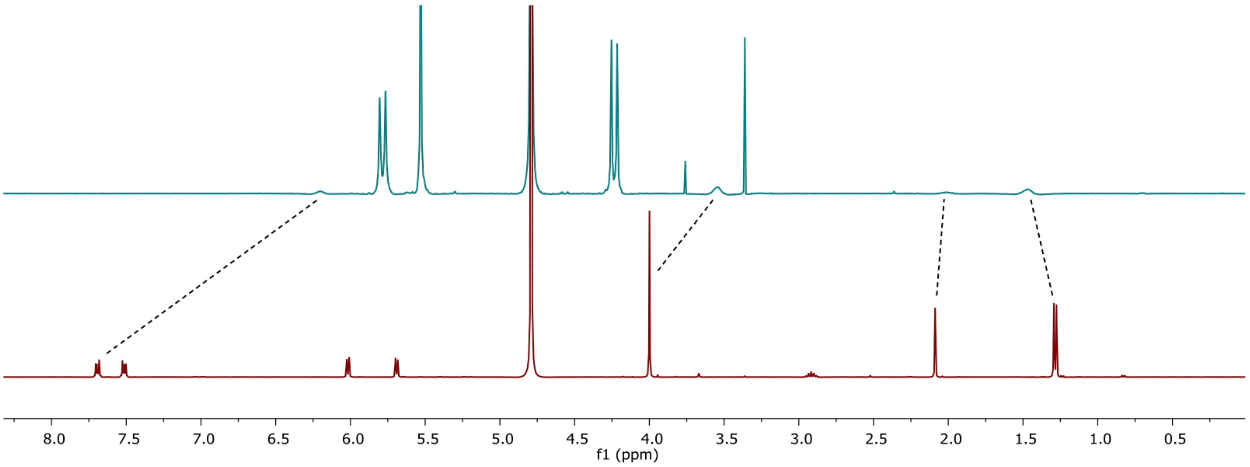

*Figure S 17: ^{1}H-NMR spectra in D_2O of **2** after treatment with $AgNO_3$ and CB[8] (top) and **2** after treatment with $AgNO_3$ (bottom).*

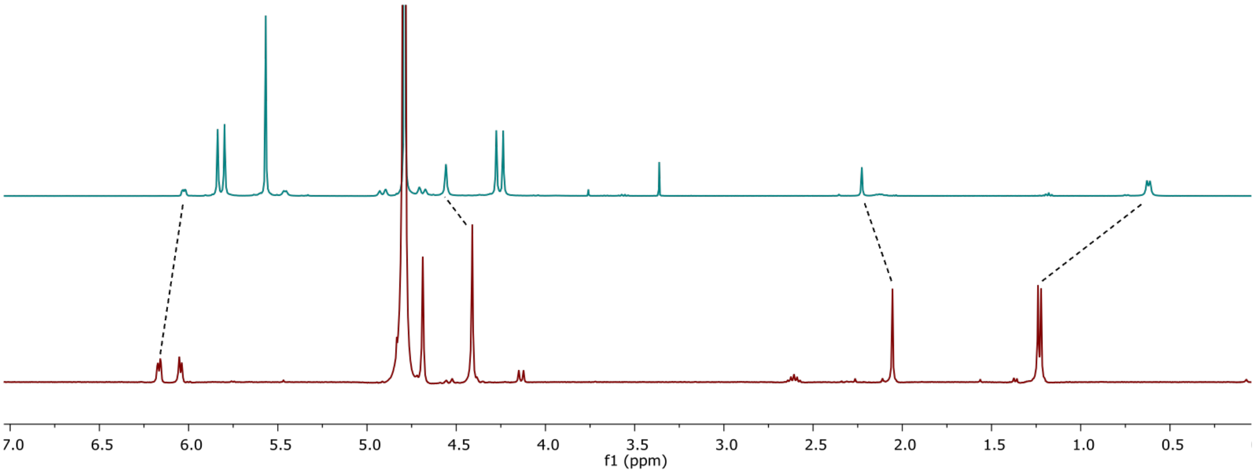

*Figure S 18: ^{1}H-NMR spectra in D_2O of **3** after treatment with $AgNO_3$ and CB[8] (top) and **3** after treatment with $AgNO_3$ (bottom).*

Mass spectrometry

Competition experiments

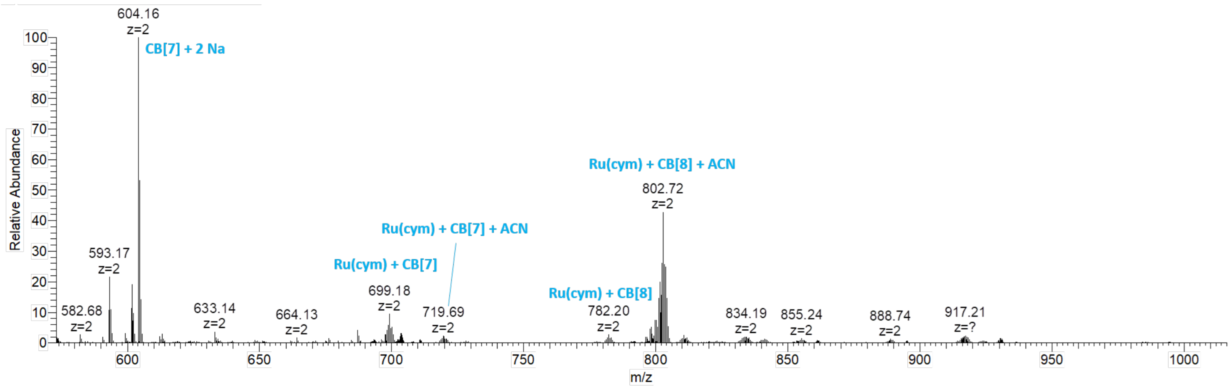

*Figure S 19: Relevant range of the full mass spectrum of a 1:1:1 mixture of complex **1** with CB[7] and CB[8].*

Table S1: Species identified by full mass spectrometry

Ruthenium complex	species	m exp.	m_{calc}	error [ppm]
	$[CB[7] + 2\ H]^{2+}$	582.1779	582.1790	-1.89
	$[CB[7] + H + Na]^{2+}$	593.1688	593.1700	-2.02
	$[CB[7] + 2\ Na]^{2+}$	604.1597	604.1610	-2.15
	$[CB[7] + Na]^{+}$	1185.3301	1185.3327	-2.19
	$[CB[8] + 2\ Na]^{2+}$	687.1840	687.1855	-2.18
**1**	$[Ru(cym)Cl + 2\ ACN]^{2+}$	159.0330	159.0331	-0.63
	$[Ru(cym)Cl]^{+}$	270.9823	270.9821	0.74
	$[Ru(cym)Cl + ACN]^{+}$	312.0087	312.0087	0.00
	$[Ru_2(cym)_2Cl_3]^{+}$	576.9337	576.9338	-0.17
	$[Ru(cym) + CB[7]]^{2+}$	699.1776	699.1789	-1.86
	$[Ru(cym) + CB[7] + ACN]^{2+}$	719.6913	719.6922	-1.25
	$[Ru(cym) + CB[8]]^{2+}$	782.2016	782.2035	-2.43
	$[Ru(cym) + CB[8] + ACN]^{2+}$	802.7170	802.7168	0.25
**2**	$[Ru(cym)(dmb)OH]^{+}$	399.0995	399.1010	-3.76
	$[Ru(cym)(dmb)Cl]^{+}$	417.0657	417.0668	-2.64
	$[Ru(cym)(dmb) + CB[7] + Na]^{3+}$	522.4761	522.4769	-1.53
	$[Ru(cym)(dmb) + CB[8] + Na]^{3+}$	577.8257	577.8268	-1.90
	$[Ru(cym)(dmb) + CB[7] + CB[8] + 2\ Na]^{4+}$	729.9525	729.9540	-2.05
	$[Ru(cym)(dmb) + CB[7]]^{2+}$	772.2195	772.2211	-2.07
	$[Ru(cym)(dmb) + CB[8]]^{2+}$	855.2438	855.2453	-1.75
	$[Ru(cym)(dmb) + CB[7] + CB[8] + Na]^{3+}$	965.2727	965.2751	-2.49
	$[Ru(cym)(dmb) + 2\ CB[8] + Na]^{3+}$	1020.6224	1020.6247	-2.25
**3**	$[Ru(cym)(pta)Cl]^{+}$	428.0581	428.0592	-2.57
	$[Ru(cym)(pta) + CB[7] + Na]^{3+}$	526.1403	526.1414	-2.09
	$[Ru(cym) + CB[7]]^{2+}$	699.1773	699.1789	-2.29
	$[Ru(cym)(pta) + CB[7]]^{2+}$	777.7155	777.7174	-2.44
	$[Ru(cym)(pta) + CB[8]]^{2+}$	860.7401	860.7420	-2.21
	$[Ru(cym)(pta) + 2\ CB[7] + Na]^{3+}$	913.5877	913.5895	-1.97

Gas phase stability by ER-MS

Table S2: Fragment ion list obtained from HCD fragmentation of the respective precursor ions

Precursor ion	species	m exp.	m_{calc}	error [ppm]
**2**@CB[7]	[dmb + H]$^{+}$	147.0913	147.0917	-2.72
	[Ru(cym)(dmb) - H]$^{+}$	381.0892	381.0904	-3.15
	[Ru(dmb) + CB[7]]$^{2+}$	699.1776	699.1789	-1.86
	[Ru(cym)(dmb) + CB[7]]$^{2+}$	772.2195	772.2211	-2.07
	[CB[7] - H]$^{+}$	1161.3326	1161.3352	-2.24
**2**@CB[8]	[dmb + H]$^{+}$	147.0913	147.0917	-2.72
	[Ru(cym)(dmb) - H]$^{+}$	381.0892	381.0904	-3.15
	[Ru(dmb) + CB[8]]$^{2+}$	788.1900	788.1909	-1.14
	[Ru(cym)(dmb) + CB[8]]$^{2+}$	855.2438	855.2453	-1.75
	[CB[8] - H]$^{+}$	1327.3815	1327.3842	-2.03
**3**@CB[7]	[Ru(cym)(pta) - $C_5H_{10}N_3$]$^{+}$	281.0024	281.0031	-2.49
	[Ru(cym)(pta) - $C_2H_4N_1$]$^{+}$	351.0554	351.0562	-2.28
	[Ru(cym) + CB[7]]$^{2+}$	699.1781	699.1789	-1.14
	[Ru(pta) + CB[7]]$^{2+}$	710.6619	710.6625	-0.84
	[Ru(cym)(pta) + CB[7]]$^{2+}$	777.7166	777.7174	-1.03
	[CB[7] + H]$^{+}$	1163.3489	1163.3508	-1.63
**3**@CB[8]	[Ru(cym)(pta) - $C_5H_{10}N_3$]$^{+}$	281.0024	281.0031	-2.49
	[Ru(cym)(pta) - $C_4H_9N_2$]$^{+}$	308.0128	308.0141	-4.22
	[Ru(cym)(pta) - $C_2H_4N_1$]$^{+}$	351.0548	351.0562	-3.99
	[Ru(cym)(pta) + H]$^{+}$	394.0968	394.0985	-4.31
	[Ru(pta) + CB[8]]$^{2+}$	793.6846	793.6874	-3.53
	[Ru(cym)(pta) + CB[8]]$^{2+}$	860.7393	860.7420	-3.14
	[CB[8] - H]$^{+}$	1327.3798	1327.3842	-3.31

Theoretical calculations

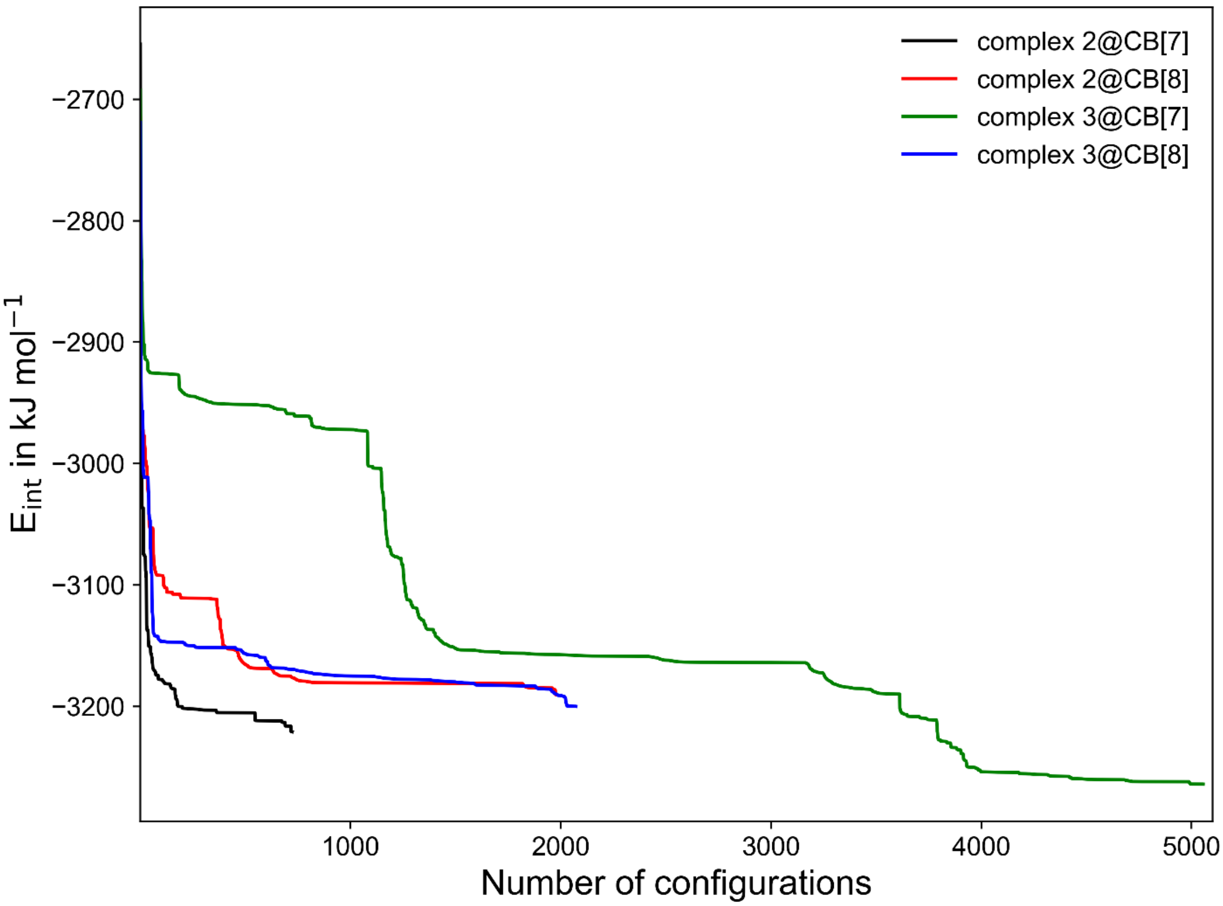

Figure S 20: Interaction energy E_{int} in kJ mol^{-1} with respect to the number of configurations obtained for the different guest@CB[n] (n = 7, 8) complexes at GFN2-xTB level of theory sorted in decreasing order. The variation in the number of configurations is due to the difference in size, resulting in a different number of accepted initial configurations.

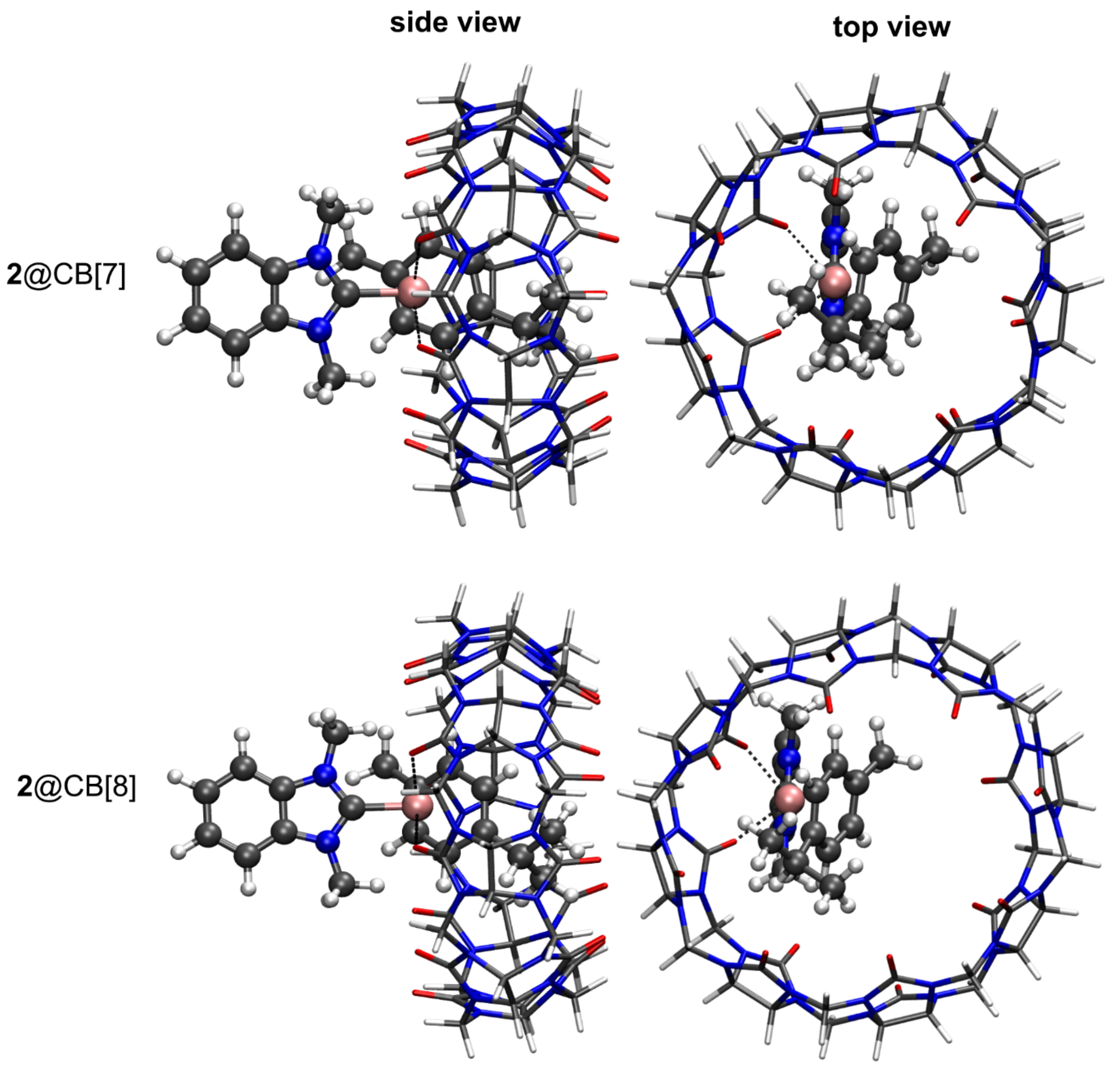

*Figure S 21: Optimized structures of **2**@CB[7] and **2**@CB[8] showing the lowest interaction energy E_{int} obtained at RIB3LYP-D3 level of theory. In both cases, the arene ligand of complex **2** is located inside the CB host system.*

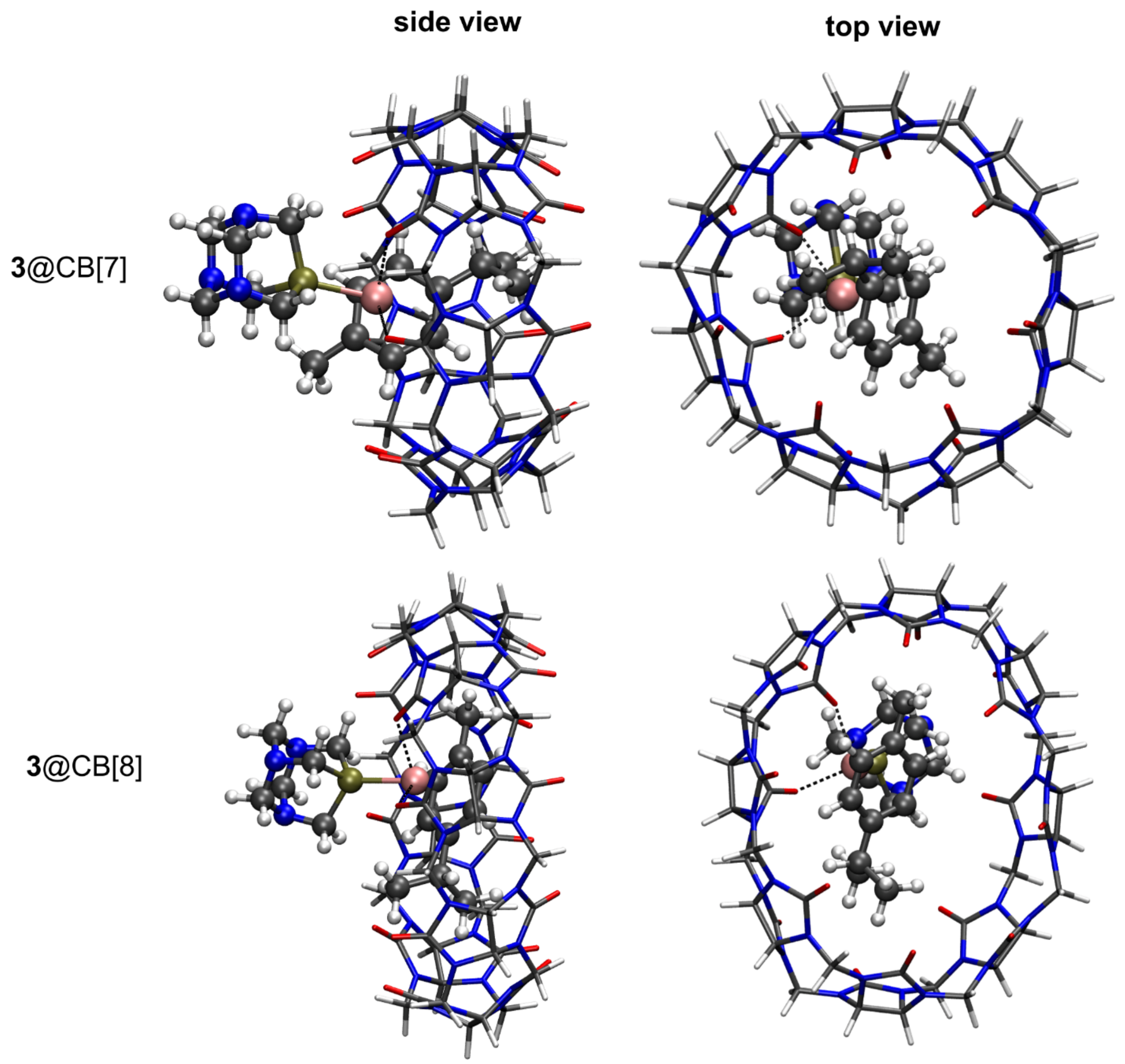

*Figure S 22: Optimized structures of **3**@CB[7] and **3**@CB[8] showing the lowest interaction energy E_{int} obtained at RIB3LYP-D3 level of theory. In both cases, the arene ligand of complex **3** is located inside the CB host system.*

*Table S3: Calculated interaction energy E_{int} in kJ.mol^{-1} obtained for the most stable host-guest compound of **2** and **3** embedded in CB[n] (n = 7,8] at RIB3LYP and RIB3LYP-D3 level of theory, respectively. The arene ligand of complexes **2** and **3** is located inside the cavity of the CB host system, as depicted in Figures S21-S22.*

Guest	Host	*E*int	
		RIB3LYP	RIB3LYP-D3
complex **2**	CB[7]	-2198.22	-2395.06
	CB[8]	-2159.71	-2331.38
	ΔE_{int}	-38.51	-63.678
complex **3**	CB[7]	-2209.11	-2402.45
	CB[8]	-2133.74	-2359.55
	ΔE_{int}	-75.37	-42.90

*Table S4: Ru-O bond length in Å in the optimized structures of **2**@CB[n] and **3**@CB[n] (n = 7, 8) at RIB3LYP and RIB3LYP-D3 level of theory, respectively. The arene ligand of complexes **2** and **3** is located outside the cavity of the CB host system, as shown in Figures 8-9.*

Guest	Host	Ru-O bond length	
		RIB3LYP	RIB3LYP-D3
complex **2**	CB[7]	2.31, 2.35	2.27, 2.31
	CB[8]	2.29, 2.34	2.29, 2.33
complex **3**	CB[7]	2.27, 2.27	2.25, 2.27
	CB[8]	2.30, 2.32	2.27, 2.34

*Table S5: RMSD in Å obtained for the most stable host-guest complex of compound **2** and **3** embedded in CB[n] (n = 7, 8) at GFN2-xTB level in comparison to respective minimum structure re-optimized at RIB3LYP and RIB3LYP-D3 level of theory. The arene ligand of complexes **2** and **3** is located outside the cavity of the CB host system.*

Guest	Host	Methods	RMSD	
			RIB3LYP	RIB3LYP-D3
complex **2**	CB[7]	GFN2-xTB	0.20	0.21
	CB[8]		0.76	0.64
complex **3**	CB[7]	GFN2-xTB	0.31	0.18
	CB[8]		0.43	0.20
